# High-flow nasal oxygenation during gastrointestinal endoscopy. Systematic review and meta-analysis

**DOI:** 10.1016/j.bjao.2022.100098

**Published:** 2022-10-18

**Authors:** Michele Carron, Enrico Tamburini, Bijan Safaee Fakhr, Alessandro De Cassai, Federico Linassi, Paolo Navalesi

**Affiliations:** 1 Department of Medicine - DIMED, Section of Anaesthesiology and Intensive Care, University of Padua, Padua, Italy; 2 Institute of Anaesthesia and Intensive Care - Azienda Ospedale Università Padova, Padua, Italy; 3 Department of Anaesthesia and Intensive Care, Ca’ Foncello Treviso Regional Hospital, Piazzale Ospedale 1,Treviso, Italy

**Keywords:** complications, digestive system endoscopy, gastrointestinal endoscopy, hypoxia, oxygen inhalation therapy

## Abstract

**Background:**

The use of high-flow nasal oxygen (HFNO) has the potential to improve patient safety by limiting hypoxaemia during gastrointestinal endoscopy. The degree of benefit is not adequately established.

**Methods:**

English language literature searches of PubMed, Scopus, Web of Science, and Cochrane Library electronic databases were performed to identify randomised controlled trials comparing HFNO and conventional oxygen therapy (COT) for patients undergoing gastrointestinal endoscopy under deep sedation. The primary endpoint was the incidence of hypoxic events observed during endoscopic procedures. The secondary endpoints were the incidence of recourse to rescue manoeuvres, procedure interruption, and adverse events. A meta-analysis and a *post hoc* trial sequence analysis were performed.

**Results:**

A total of 2867 patients from six randomised controlled trials were considered. Desaturation was observed in 5.2% and 27.2% of patients receiving HFNO and COT, respectively. Desaturation <90% was observed in 1.8% and 12.6% of the patients receiving HFNO and COT, respectively. In the subgroup analysis, desaturation occurrence was lower during HFNO than during COT in non-obese patients (2.2% *vs* 25.2%) and obese patients (22.9% *vs* 43.3%). Desaturation occurrence was lower during maximum (3.6% *vs* 26.9%) and minimum (15.9% *vs* 29.8%) HFNO therapy than during COT. HFNO showed a lower recurrence to rescue manoeuvres rate (4.7% *vs* 34.3%), a lower procedure interruption rate (0.4% *vs* 6.7%), and a lower adverse events rate (18.7% *vs* 21%) than COT. A high level of heterogeneity between the studies precluded confidence in drawing inference from the meta-analysis.

**Conclusions:**

The evidence reviewed suggests that compared with COT, HFNO has fewer hypoxaemic events during gastrointestinal endoscopy, but this may not apply to all patients and clinical scenarios.

High-flow nasal oxygen (HFNO) therapy provides warmed, humidified gases at flows of up to 60–70 L min^−1^, with inspired oxygen concentrations of up to 100%. It is considered to have a number of physiological advantages compared with conventional oxygen therapy (COT). Warmed, humidified gas enhances mucociliary clearance, prevents airway surface dehydration, and generally improves comfort. The high gas flow generates a reservoir of oxygen, maintains constant fractional inspired oxygen (FiO_2_, close to 1.0), reduces dead space, and increases carbon dioxide (CO_2_) washout. The positive airway pressure (mean values ranging between 2.7 and 7.4 cm H_2_O) increases end-expiratory lung volume and alveolar recruitment and decreases atelectasis.^1^, ^2^, ^3^ HFNO is increasingly used as part of both the ward-based and the critical care management of respiratory failure to treat hypoxaemia in acute respiratory failure.^1^, ^2^, ^3^ HFNO has been proposed to limit oxygen desaturation by prolonging apnoeic oxygenation during tracheal intubation in both ICU and operating theatres.^1^, ^2^, ^3^

All sedative drugs commonly used in gastrointestinal endoscopy can lead to a dose-dependent depression of airway dilator muscle activity and respiratory control centres (with a decrease in depth, rate, or both of ventilation), and may be associated with negative inotropic effects and peripheral vasodilation.^4^^,^^5^ Hypoxaemia is the most common adverse event that occurs during sedation, no matter which sedative agent is used.^6^, ^7^, ^8^ It can occur in 26–85% of cases and results from a combination of airway obstruction by the endoscope, anaesthesia-induced upper airway collapse, respiratory depression, and lung compression because of intestinal gas insufflation.^9^, ^10^, ^11^, ^12^, ^13^, ^14^ Older or obese patients or those in a high ASA physical status class are at particular risk of hypoxaemia and potentially prone to hypoxaemia-induced complications.^4^^,^^6^^,^^12^ If severe or prolonged, hypoxia can lead to serious adverse events, such as permanent neurological damage, myocardial ischaemia, cardiac arrhythmia, cardiorespiratory arrest, or even death.^6^^,^^7^ Therefore, it is important to take steps to reduce the incidence of hypoxia and severe hypoxia during sedated endoscopy procedures. For this reason, supplemental oxygen should be available whenever sedation is used, especially in long procedures or whenever a hypoxic period is anticipated.^4^ There is a growing body of evidence that the use of HFNO gastrointestinal endoscopy may improve safety by avoiding or limiting hypoxaemia. However, there is a need to understand the degree of benefit it confers and which patients any benefits may apply to.

## Methods

The Preferred Reporting Items for Systematic Reviews and Meta-Analyses (PRISMA) 2020 guidelines were used to prepare this manuscript.^15^ The study protocol was registered in PROSPERO (reference: CRD42020178829) on 5 July 2020.

Randomised controlled trials (RCTs) comparing HFNO and COT (via a nasal cannula or face mask) of patients undergoing gastrointestinal endoscopy (upper, lower, or both) under deep sedation were considered. The inclusion criteria for studies analysed in this meta-analysis were as follows: HFNO use, COT via a nasal cannula or face mask, English language, and adult patients (≥18 yr). The following exclusion criteria were used: observational, non-clinical, or paediatric studies; lack of data or a full-text version of the article; and non-peer-reviewed articles.

We searched the PubMed, Scopus, Web of Science, and Cochrane Library electronic databases, limiting the search to articles published from 1 January 2000 to 30 June 2021. Using the “AND” function, the Medical Subject Headings (MeSH) term ‘oxygen inhalation therapy’ was combined with the following MeSH terms: ‘digestive system endoscopy’ OR ‘endoscopy, gastrointestinal.’ To improve the search accuracy, searches were repeated using ‘high-flow nasal oxygen’ OR ‘high-flow nasal oxygen therapy’ OR ‘high-flow nasal cannula’ OR ‘high-flow nasal cannula therapy’ OR ‘HFNO’ OR ‘HFNOT’ OR ‘HFNC’ OR ‘HFNCT’ instead of the MeSH term ‘oxygen inhalation therapy.’ We also checked the reference lists of the evaluated studies. The following search filters were used: ‘controlled clinical trial,’ ‘randomized controlled trial,’ ‘adult,’ and ‘English language.’ The search strategy is reported in the Supplementary Material (SM) 1.

Two authors independently screened the titles and abstracts of articles retrieved by the search strategies based on MeSH terms (ET, BSF) and non-MeSH terms (FL, ADC) and excluded non-relevant articles. The full texts of the remaining studies were then assessed to determine whether they met the pre-determined selection criteria. Using pre-designed data collection forms, data were extracted independently by two authors (ET, BSF) for the MeSH search and by two authors (FL, ADC) for the non-MeSH search from the included studies. An author not involved in the literature search (MC) resolved any discrepancies arising during the study selection, data extraction, or trial evaluation process.

The primary endpoint was the incidence of hypoxic events defined as ‘desaturation’ (decreased peripheral oxygen saturation [SpO_2_]) according to the studies' endpoints observed after the induction and maintenance of sedation for endoscopic procedures. A subgroup analysis evaluated only hypoxic events defined by SpO_2_ <90%. The primary outcome was further evaluated by comparing COT with both maximum and minimum HFNO therapy, HFNO in non-obese and in obese patients, HFNO in short and in long duration procedures, and HFNO during sedation and during sedation plus analgesia with opioids. A maximum HFNO therapy was defined by a flow rate of ≥40 L min^−1^ during HFNO. A minimum HFNO therapy was defined by a flow rate of <40 L min^−1^ during HFNO.^2^^,^^3^ Obesity was defined by a body mass index (BMI) of ≥30 kg m^−2^ according to the World Health Organization's definition. When the patients were not stratified based on BMI, non-obesity was defined by a 75 percentile of <30 kg m^−2^. A short endoscopic procedure was defined by a duration time of ≤10 min.^1^, ^2^, ^3^

The secondary endpoints were the incidence of recourse to rescue manoeuvres, procedure interruption, and adverse events. The rescue manoeuvres were all interventions by the anaesthetist necessary to correct ‘desaturation’ according to the studies' endpoints or to maintain patency of the airway. These interventions were divided into ‘minor’ interventions (an intervention that did not require using further devices [e.g. jaw lift, need for modification of oxygen flow settings]) and ‘major’ interventions (e.g. bag-mask ventilation, placement of any device [e.g. nasopharyngeal airway, tracheal tube], or recourse to any invasive procedures [e.g. emergency front-of-neck access] to secure the airway). Adverse events were regarded as total adverse events and specific adverse events, which were divided into neurological adverse events (e.g. myoclonus, agitation on awakening, increased tone with twitching and rhythmic movements not perceived as generalized tonic-clonic seizures, opisthotonos, involuntary movements, or epilepsy), respiratory adverse events (any airway- or respiratory-related adverse event, with or without desaturations [e.g. airway obstruction, apnoea or bradypnoea episodes, hypercapnia]), cardiovascular adverse events (e.g. arrhythmia, bradycardia, hypotension, or the use of vasopressor), gastrointestinal or abdominal adverse events (e.g. nausea, vomiting, or abdominal pain), and other serious adverse events (e.g. ICU admission, prolonged hospitalisation, cardiac arrest, death).

The authors involved in the literature search (BSF, ET, FL, ADC) independently evaluated the quality of the included RCTs by using the Risk of Bias (RoB) 2 Tool.^16^ The RoB 2 Tool features five risk-of-bias domains (randomisation process, deviations from intended interventions, missing outcome data, measurement of the outcome, selection of the reported result). Within each domain, a series of questions (‘signalling questions’) aim to elicit information about the features of the trial that are relevant for the risk of bias. A proposed judgement about the risk of bias arising from each domain is generated by an algorithm, based on answers to the signalling questions. Judgement can be a ‘low’ or ‘high’ risk of bias or can express ‘some concerns’.^16^ Disagreements were resolved by discussion with a further author (MC).

The Grades of Recommendation, Assessment, Development and Evaluation (GRADE) approach was used to assess the certainty of evidence related to the primary outcome.^17^ Starting from ‘high quality’ of evidence, the certainty of evidence for each outcome was downgraded by one level for serious, or by two levels for very serious study limitations, such as risk of bias, indirectness of evidence, inconsistency, imprecision of effect estimates, or other considerations. ‘Indirectness of evidence’ was considered when subjects, intervention, or outcome were different from those of primary interest for the meta-analysis. ‘Inconsistency of the outcome’ was assessed considering the following: (i) wide variance of point estimates across studies; (ii) minimal or no overlap of confidence intervals (CI); and (iii) large *I*^2^ statistic, which quantifies the proportion of the variation in point estimates as a result of among-study differences. ‘Imprecision of effect’ occurred in case of small sample size, number of events, and uncertainty about magnitude of effect given by large intervals of confidence. ‘Other considerations’ include publication bias, large effect, plausible confounding, and dose response gradient.^17^

### Statistical analyses

Frequency statistics were used to count the number of times that each variable occurred according to each study's endpoint.

The meta-analysis was performed within a frequentist framework using both random and fixed effects models, computing the relative risk (RR) and 95% CI for binary outcome data. The Mantel–Haenszel method was used to calculate the fixed effects estimate for dichotomous data. When the RR was calculated, 0.5 was added to the frequencies of all studies with a zero number of events. The random effects model was computed with inverse-variance weighting using the DerSimonian and Laird method to account for heterogeneity. Heterogeneity across studies was tested using the *I*^2^ statistic.^18^ A threshold of *P*<0.1 was used to determine whether heterogeneity was present. *I*^2^ was considered low (<25%), moderate (25–50%), or high (>50%).^18^ The random effects model was preferred for the final analysis. If the number of included studies was very small, then it would be impossible to estimate the between-studies variance (tau-squared) with any precision. In this case, the results of the *Q* test will be used to identify substantial heterogeneity. A funnel plot was used to estimate the effect (log risk ratio) from individual studies against standard errors. Outliers resulting from leave-one-out diagnostics were evaluated to establish which studies had a strong influence on the results (as reflected, for example, by their Cook's distances).

A trial sequential analysis (TSA) was performed.^19^ TSA is a cumulative meta-analysis method used to weigh type I and II errors and to estimate when the effect is large enough to be unaffected by further studies.^19^ The required sample sizes for the calculated minimal intervention effects were estimated considering a type I error of 5% and a power of 80%. The choice of *a priori* RR reduction (RRR) was based on the analysis of the low-risk-of-bias trials, by excluding the high-risk-of-bias studies, which could overestimate the intervention effect.^19^

The statistical analysis was carried out using R (Free Software supported by R Foundation for Statistical Computing, Vienna, Austria), version 4.1.0. Specifically, TSA computations were implemented making use of the libraries ‘ldbounds’ and ‘rpact.’ All *P*-values were two-tailed, with statistical significance set at <0.05.

## Results

### Paper selection

**Fig 1:**
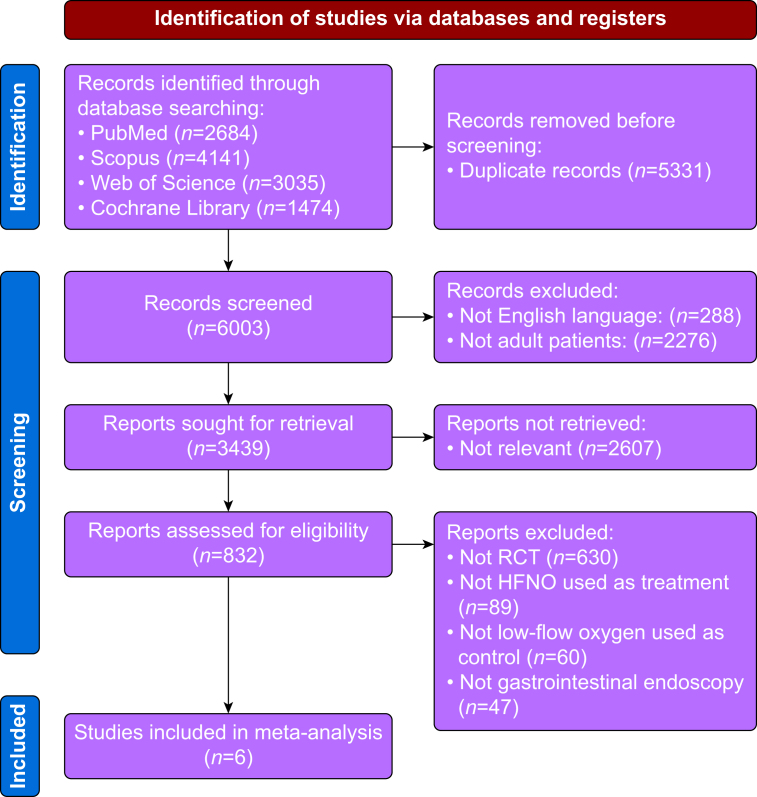
The Preferred Reporting Items for Systematic Reviews and Meta-Analyses (PRISMA) flow diagram of the study selection process. HFNO, high-flow nasal oxygen; RCT, randomized controlled trials.

Of the 6003 reports initially identified by screening the literature, 5997 records were excluded because they did not meet the inclusion criteria. Therefore, six RCTs involving a total of 2867 patients were eligible for meta-analysis.^9^, ^10^, ^11^, ^12^, ^13^, ^14^ The PRISMA flow diagram of our study selection protocol is presented in Fig. 1. The characteristics of the included RCTs and the main results are detailed in Table 1, Table 2, respectively.

**Table 1:** Characteristics of studies considered for review and meta-analysis. Data and *P*-values are from recent studies.^9^, ^10^, ^11^, ^12^, ^13^, ^14^

Study (yr)	Procedure	Anaesthetic drugs	Population (COT/HFNO)	COT	HFNO	Primary endpoint
Teng and colleagues^9^ (2019)	OGDS	Propofol (TCI), midazolam, alfentanil	General (51/50)	5 L min^−1^ NC	100% FiO_2_ flow rate 30 L min^−1^	Hypoxaemia (AUC of SpO_2_ <90%)
Lin and colleagues^10^ (2019)	Gastroscopy	Propofol (push strategy)	General (1000/994)	2 L min^−1^ NC	100% FiO_2_ flow rate 60 L min^−1^	Hypoxaemia (75% <SpO_2_<90% for <60 s)
Riccio and colleagues^11^ (2019)	Elective colonoscopy	Propofol	Obese (31/28)	4 L min^−1^ NC	36–40% FiO_2_ flow rate 60 L min^−1^	Hypoxaemia (SpO_2_ <90%)
Nay and colleagues^12^ (2021)	Gastrointestinal endoscopy (upper, lower, both)	Propofol, midazolam, opioids, ketamine,	General (188/191)	0.50 FiO_2_ NC or mask	50% FiO_2_ flow rate 70 L min^−1^	Hypoxaemia (SpO_2_ ≤92%)
Kim and colleagues^13^ (2021)	ERCP (prone position)	Propofol, midazolam	General (36/36)	5 L min^−1^ NC	100% FiO_2_ flow rate 50 L min^−1^	Hypoxaemia (lowest oxygen saturation measured as SpO_2_)
Mazzeffi and colleagues^14^ (2021)	OGDS	Propofol, midazolam, fentanyl	General (130/132)	6 L min^−1^ NC	100% FiO_2_ flow rate 20 L min^−1^	Hypoxaemia (SpO_2_ <92% for ≥15 s)

AUC, area under the curve; COT, conventional oxygen therapy; ERCP, endoscopic retrograde cholangiopancreatography; FiO_2_, fraction of inspired oxygen; HFNO, high-flow nasal oxygen; NC, nasal cannula; OGDS, oesophagogastroduodenoscopy; SpO_2_, peripheral saturation of oxygen; TCI, target-controlled infusion.

**Table 2:** Outcomes of studies considered for review and meta-analysis.

Study (yr)	Population COT/HFNO∗	Total hypoxic events COT/HFNO (%)	*P*-value	Total rescue manoeuvres COT/HFNO (%)	*P*-value	Total adverse events COT/HFNO (%)	*P*-value	Procedure interruption COT/HFNO (%)	*P*-value
Teng and colleagues^9^ (2019)	General (51/50)	11 (21.6)/1 (2.0)	0.004	9 (17.6)/1 (2.0)	0.006	38 (74.5)/44 (88)	NA	—	—
Lin and colleagues^10^ (2019)	General (1000/994)	253 (25.3)/16 (1.6)	<0.001	319 (31.9)/8 (0.8)	<0.001	107 (10.7)/57 (5.7)	NA	—	—
Riccio and colleagues^11^ (2019)	Obese (31/28)	14 (45.2)/11 (39.3)	0.79	16 (51.6)/15 (53.6)	NA	—	—	—	—
Nay and colleagues^12^ (2021)	General (188/191)	63 (33.5)/18 (9.4)	<0.001	115 (61.2)/39 (20.4)	<0.001	44 (23.4)/32 (16.8)	NA	5 (2.7)/1 (0.5)	0.12
Kim and colleagues^13^ (2021)	General (36/36)	7 (19.4)/0 (0.0)	0.011	30 (83.3)/0 (0.0)	NA	—	—	10 (27.8)/0 (0.0)	0.001
Mazzeffi and colleagues^14^ (2021)	General (130/132)	43 (33.1)/28 (21.2)	0.03	—	—	99 (76.2)/123 (93.2)	NA	—	—

COT, conventional oxygen therapy; HFNO, high-flow nasal oxygen; NA, not applicable.

∗General population included both non-obese and obese patients. Obesity: body mass index ≥30 kg m^−2^.

Two populations of patients, non-obese and obese patients, were analysed separately for the primary outcome.

### Risk of bias assessment

**Fig 2:**
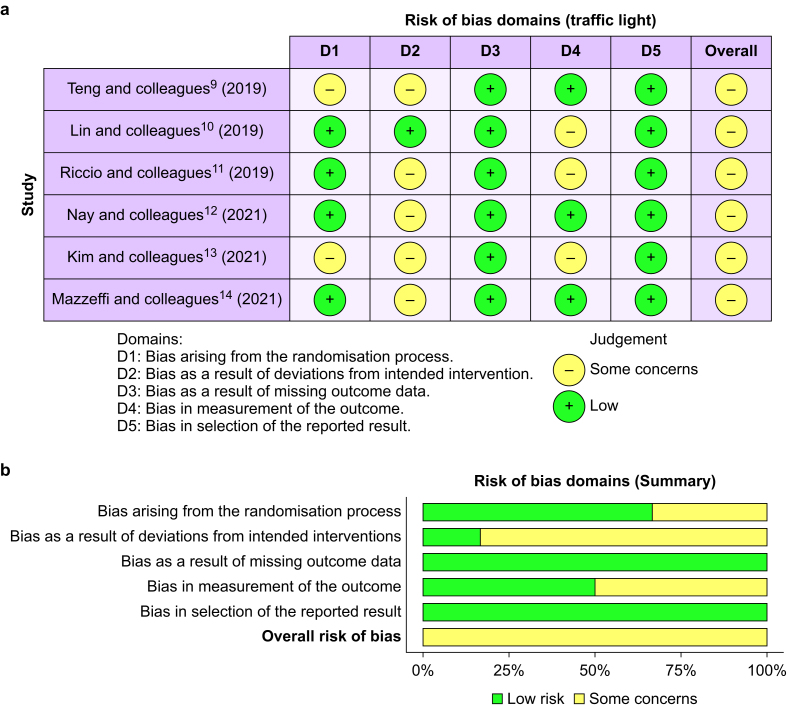
Risk of Bias (RoB) 2 assessment (traffic light [a] and summary [b]) of included RCTs. The graphs of the Risk of Bias (RoB) 2 assessment show that the included studies were subject to an overall unclear risk of bias. Details are available in the Supplementary material (SMc2).

The RoB 2 assessment of the included RCTs is shown in Fig. 2, which shows that the included studies were subject to an overall unclear risk of bias. All studies provided information on the randomisation of patients,^9^, ^10^, ^11^, ^12^, ^13^, ^14^ but two RCTs did not specify the allocation concealment or masking strategy used.^9^^,^^13^ Only one study specified the method for blind operators and participants.^10^ All studies reported outcome data according to the endpoint of the study.^9^, ^10^, ^11^, ^12^, ^13^, ^14^ Only in three studies was the outcome assessor unaware of the intervention received by study participants.^9^^,^^12^^,^^14^ The risk of reporting bias was low in all studies.^9^, ^10^, ^11^, ^12^, ^13^, ^14^ Outcome measurements and analyses were conducted in accordance with pre-specified intentions to eliminate the possibility of result selection.^9^, ^10^, ^11^, ^12^, ^13^, ^14^ The detailed reasons for the risk of bias judgements are available in the SM 2.

### Outcomes

#### Primary endpoint

The incidence of desaturation was compared in 1431 patients receiving HFNO with 1436 patients receiving COT (Table 3).^9^, ^10^, ^11^, ^12^, ^13^, ^14^ Desaturation was observed in 5.2% and 27.2% of all patients receiving HFNO and COT, respectively (Table 3). With the exception of one study,^11^ the advantage of HFNO over COT in reducing hypoxaemic events was statistically significant.^9^^,^^10^^,^^12^, ^13^, ^14^ The certainty of evidence was evaluated as high for the primary outcome (SM3). The incidence of SpO_2_<90% in patients receiving HFNO (*n*=1299) was 1.8% compared with 12.6% of patients receiving COT (*n*=1306) (Table 3).^9^, ^10^, ^11^, ^12^, ^13^ The difference in the incidence of desaturation between the two approaches observed in all patients was confirmed in both non-obese and obese patients evaluated separately (Table 3).

**Table 3:** Incidence of hypoxic events, rescue treatment, procedure interruption, and adverse events.

General population of patients∗	HFNO *n*/*N* (%)	COT n/N (%)
Hypoxic events ^(^9, 10, 11, 12, 13, 14^)^	74/1431 (5.2)	391/1436 (27.2)
-hypoxic events (SpO_2_ <90%) ^(^9, 10, 11, 12, 13^)^	23/1299 (1.8)	165/1306 (12.6)
Hypoxic events with HFNO ≥40 L min^−1^^(^10, 11, 12, 13^)^	45/1249 (3.6)	337/1255 (26.9)
Hypoxic events with HFNO <40 L min^−1^^(^9, 10, 11, 12, 13, 14^)^	29/182 (15.9)	54/181(29.8)
Hypoxic events in short procedure ^9^^,^^10^	17/1044 (1.6)	264/1051 (25.1)
-hypoxic events in short procedure (SpO_2_ <90%) ^9^^,^^10^	1/1044 (0.1)	101/1051 (9.6)
Hypoxic events in long procedure ^(^11, 12, 13, 14^)^	57/387 (14.7)	127/385 (33)
-hypoxic events in long procedure (SpO_2_ <90%) ^(^11, 12, 13^)^	22/255 (8.6)	64/255 (25.1)
Rescue treatment ^(^9, 10, 11, 12, 13, 14^)^	67/1431 (4.7)	492/1436 (34.3)
-minor rescue treatment ^(^10, 11, 12, 13^)^	57/1249 (4.6)	465/1255 (37.1)
-major rescue treatment ^(^10, 11, 12, 13, 14^)^	9/1381(0.7)	18/1385 (1.3)
Procedure interruption ^12^^,^^13^	1/227 (0.4)	15/224 (6.7)
Total adverse events ^9^^,^^10^^,^^12^^,^^14^	256/1367 (18.7)	288/1369 (21)
-respiratory no-hypoxaemic events ^9^^,^^10^^,^^12^^,^^14^	128/1367 (9.4)	120/1369 (8.8)
-cardiovascular events ^9^^,^^10^^,^^12^^,^^14^	111/1367 (8.1)	119/1369 (8.7)
--bradycardia ^10^^,^^12^	17/1168 (1.4)	28/1160 (2.4)
--hypotension^10^^,^^12^^,^^14^	83/1317 (6.3)	99/1318 (6.7)
Hypoxic events with sedation ^10^^,^^11^	11/1022 (1.1)	104/1031 (10.1)
Hypoxic events with sedation and opioid ^9^^,^^13^	1/86 (1.2)	18/87 (20.7)
**Non-obese patients**		
Hypoxic events ^9^^,^^10^^,^^12^^,^^13^	27/1216 (2.2)	309/1226 (25.2)
-hypoxic events (SpO_2_ <90%) ^9^^,^^10^^,^^12^^,^^13^	5/1216 (0.4)	132/1226 (10.8)
Rescue treatment ^9^^,^^10^^,^^13^	9/1080 (0.8)	358/1087 (32.9)
-minor rescue treatment ^10^^,^^13^	8/1030 (0.8)	346/1036 (33.4)
-major rescue treatment ^10^^,^^13^	0/1030 (0.0)	3/1036 (0.3)
Total adverse events ^9^^,^^10^	101/1044 (9.7)	145/1051 (13.8)
-respiratory non-hypoxaemic events ^9^^,^^10^	47/1044 (4.5)	48/1051 (4.6)
-cardiovascular events ^9^^,^^10^	37/1044 (3.5)	48/1051 (4.6)
**Obese patients**		
Hypoxic events ^11^^,^^12^	19/83 (22.9)	39/90 (43.3)
-hypoxic events (SpO_2_ <90%) ^11^^,^^12^	18/83 (21.7)	33/90 (36.7)

COT, conventional oxygen therapy; HFNO, high-flow nasal oxygen.

∗Hypoxic events were defined as ‘desaturation’ (decreased peripheral capillary oxygen saturation [SpO2]) according with the studies' endpoints observed after the induction and the maintenance of sedation for gastrointestinal endoscopy. A subgroup analysis evaluated hypoxic events defined as SpO2 <90%. General population included both non-obese and obese patients. Obesity: body mass index ≥30 kg m^−2^.

In subgroup analyses, maximum HFNO therapy (3.6% *vs* 26.9%)^10^, ^11^, ^12^, ^13^ and minimum HFNO therapy (15.9% *vs* 29.8%)^9^^,^^14^ demonstrated a lower incidence of desaturation compared with COT. HFNO showed a lower incidence of desaturation than COT in short^9^^,^^10^ and long^11^, ^12^, ^13^, ^14^ procedures, and when using sedation alone^10^^,^^11^ or in combination with opioids (Table 3).^9^^,^^13^

### Secondary endpoints

Overall, 67 of 1431 patients (4.7%) who received HFNO and 492 of 1436 patients (34.3%) who received COT required rescue manoeuvres.^9^, ^10^, ^11^, ^12^, ^13^, ^14^ The use of both minor and major rescue manoeuvres was less during HFNO than during COT (Table 3). HFNO showed an overall lower rate of procedure interruption than COT (0.4% *vs* 6.7%) (Table 3).^12^^,^^13^

Overall, 18.7% of patients receiving HFNO and 21% of those receiving COT suffered from adverse events (Table 3).^9^^,^^10^^,^^12^^,^^14^ Non-hypoxaemic respiratory adverse events occurred in 9.4% of patients receiving HFNO and 8.8% of patients receiving COT (Table 3).^9^^,^^10^^,^^12^^,^^14^ One of 1431 (6.98 per 10 000) patients receiving HFNO therapy and 2 of 1436 (13.92 per 10 000) patients receiving COT required tracheal intubation. One study reported a lower mean value of end-tidal CO_2_ at the end of the procedure in patients receiving HFNO group than in those receiving COT (30.4 mm Hg *vs* 33.9 mm Hg, *P*=0.045).^13^ A further study reported higher mean maximum values of transcutaneous blood CO_2_ in the HFNO group than in the COT group of patients (54.3 mm Hg *vs* 51.5 mm Hg, *P*=0.04).^14^ Overall, 8.1% of patients receiving HFNO and 8.7% of those receiving COT experienced cardiovascular adverse events.^9^^,^^10^^,^^12^^,^^14^ Among cardiovascular adverse events, tachycardia,^10^ bradycardia,^10 12^ and hypotension^10^^,^^12^^,^^14^ occurrence was lower with HFNO than with COT (Table 3). In one study, HFNO was associated with fewer neurological adverse events (1.1% *vs* 3.4%) and more abdominal adverse events (1.5% *vs* 0.6%) than COT.^10^ No cases of perioperative cardiac arrest or death associated with HFNO therapy or COT were reported.

### Meta-analyses

The meta-analyses revealed a high degree of heterogeneity, limiting the interpretation of the results and precluding sound inference. The results of the meta-analyses and forest plots are reported in SM4 and SM5, respectively. Funnel plots and charts of highly influential studies (red points) are reported in SM6. The results of the TSA and TSA graphs are reported in SM7 and SM8, respectively.

## Discussion

Compared with COT, HFNO, which provides a high and stable FiO_2_ and a warmed, humidified high gas flow, has the potential for reducing the occurrence of hypoxic events, rescue manoeuvres, and procedure interruption in patients undergoing sedation for gastrointestinal endoscopy. This may not apply to all patients and clinical scenarios. There are conflicting findings regarding the use of HFNO in obese patients, with descriptive analysis yielding significant benefits in some studies but not others. The subgroup analysis revealed that maximum HFNO therapy was superior to minimum HFNO therapy, and the advantage of using HFNO compared with COT is greater during sedation plus analgesia with opioids than sedation alone. Beyond hypoxaemic events, the effect of HFNO on adverse events appeared to be minor.

Supplemental oxygen is required to prevent or treat hypoxaemia during sedation for gastrointestinal endoscopy.^4^^,^^6^^,^^7^ HFNO may better prevent hypoxaemia by enabling a higher FiO_2_ than is possible with COT, up to 1.0, as reported in different RCTs,^9^^,^^10^^,^^13^^,^^14^ where the estimated FiO_2_ during COT was significantly lower, around 0.3,^10^ 0.4,^9^^,^^13^ or 0.45.^14^ When a similar FiO_2_ (i.e. FiO_2_ 0.5) was used, including patients with a BMI of ≥30 kg m^−2^,^12^ HFNO was shown to significantly reduce the likelihood of hypoxaemic adverse events compared with COT.^12^ However, these benefits were not confirmed when considering only those patients with a BMI of ≥40 kg m^−2^.^11^

Factors other than higher FiO_2_ need, therefore, to be considered. HFNO may prevent hypoxaemia by providing positive airway pressure, which increases the end-expiratory lung volume and thus alveolar recruitment.^9^, ^10^, ^11^, ^12^, ^13^, ^14^ Positive nasopharyngeal pressure—mainly determined by the volume of the constant flow, which can be increased up to 60–70 L min^−1^, and by the expiratory flow of the patient—increases as the flow increases.^20^^,^^21^ This was observed when the patient's mouth is either closed or open, although the pressure is higher with the former than with the latter.^21^ It has been estimated that for every 10 L min^−1^ increase in gas flow, the mean nasopharyngeal pressure increases by 0.69 cm H_2_O with the mouth closed and by 0.35 cm H_2_O with the mouth open.^21^ All the studies included in our analysis used a flow rate higher in the HFNO group than in the COT group, from 20^14^ or 30^9^ to 50,^13^ 60,^10^^,^^11^ or 70^12^ L min^−1^. With the exception of one study involving morbidly obese patients, HFNO was shown to significantly reduce the incidence of hypoxaemia compared with COT in these studies by ∼12%,^14^ 18%,^9^ 19%,^13^ and 24%,^10^^,^^12^ respectively. Our analysis revealed that maximum HFNO therapy is more effective than minimum HFNO therapy in reducing hypoxic events.

The flow rate was also the dominant factor governing CO_2_ concentration. The average CO_2_ concentration decreased with increasing cannula flow rate.^20^ While a low flow rate (i.e. 20 L min^−1^) during HFNO was associated with no differences in the incidence of hypercarbia,^14^ a higher flow rate (i.e. 50 L min^−1^) during HFNO resulted in a significantly lower mean end-tidal CO_2_ value at the end of the procedure compared with COT.^13^ Apart from counteracting hypercapnia, the CO_2_ washout effect provides an anatomical oxygen reservoir within the nasopharynx and oropharynx, reducing dead space and, in turn, the work of breathing that may be increased in patients suffering from acute respiratory failure.^23^ Even though different mechanisms may be involved, the study by Nay and colleagues^12^ showed that the beneficial effect of HFNO seems predominantly attributable to its positive nasopharyngeal pressure, dead-space washout effects, or both.^12^

The HFNO benefits seem to be limited by certain conditions, such as concomitant opioid use and morbid obesity, which, in conjunction with the sedative drugs, negatively affect the airways' patency and lung ventilation. Among the advantages of HFNO, positive nasopharyngeal pressure is essential for decreasing upper airway obstruction and improving ventilation by recruiting and stabilising previously collapsed alveoli, thus increasing lung volumes. This may justify the benefits of HFNO observed in non-obese and obese patients^12^ although not in morbidly obese patients.^11^ The use of an FiO_2_ of 1.0 rather than 0.3, may increase atelectasis. Atelectasis causes shunting of blood through non-ventilated lung tissue and oxygenation impairment.^22^ So, it is theoretically possible that HFNO with an FiO_2_ of 1.0 may not improve or, potentially, may worsen hypoxaemia (and hypercapnia) in morbidly obese patients.^11^ However, considering that both studies involving obese patients used an FiO_2_ of 0.36–0.4^11^ or 0.5,^12^ other factors should be considered to explain the different outcomes.^11^^,^^12^ Obesity and, particularly, central adiposity were shown to increase pharyngeal collapsibility,^23^ affect lung volumes,^24^ increase atelectasis,^25^ and decrease oxygen levels,^26^ with larger changes as the BMI increased.^23^, ^24^, ^25^, ^26^ So, morbid obesity predisposes patients to upper airway obstruction,^23^ alveolar hypoventilation,^24^^,^^27^ increased atelectasis,^26^^,^^27^ hypoxaemia, and hypercapnia under sedation or anaesthesia,^28^, ^29^, ^30^ to an extent that potentially counteracts the benefits of HFNO.

Hypoxia is the most common respiratory adverse event that occurs during sedation that may have an impact on outcome. The analysis of RCTs showed that the incidence of tracheal intubation for a respiratory adverse event was approximately 7 per 10 000 HFNO cases and 14 per 10 000 COT cases. In retrospective analyses, Goudra and colleagues^6^^,^^7^ reported an incidence of tracheal intubation of 6.06 per 10 000 cases (17 of 28 008), whereas Agostoni and colleagues^8^ reported 7.41 per 10 000 (13 of 17 542). Our review of RCTs did not reveal any patients who died or had a cardiac arrest, although such cases are reported in the literature. In retrospective analyses of propofol sedation for gastrointestinal procedures, the incidence of cardiac arrest was 6.06 per 10 000 (17 of 28 008)^7^ and 3.99 per 10 000 (seven of 17 542),^8^ whereas the incidence of death was 4.28 per 10 000 (12 of 28 008)^7^ and 1.7 per 10 000 (three of 17 542),^8^ respectively.

We acknowledge some limitations of this work. First, the number of included studies is limited. Further data are required to support the benefits of HFNO compared with COT in improving outcomes, particularly in high-risk patients, such as those suffering from morbid obesity. Second, heterogeneity across studies was considerable for the outcomes considered. Significant statistical heterogeneity suggests an inconsistency among estimates that needs to be addressed. However, exploring this heterogeneity was not possible because of the limited number of included studies. Third, the difficulty in blinding operators and outcome assessors during many studies may introduce bias. However, no apparent imbalances are reported when comparing HFNO with COT. Finally, the economic aspects of the treatments were not considered. HFNO therapy is more expensive than COT,^10^ which may prevent its widespread use during gastrointestinal endoscopy. For this reason, HFNO has first been suggested for patients who have a higher basal risk of hypoxaemia than the general population.^12^ Regarding this, additional data are necessary for those with morbid obesity. No data in support of a more favourable cost–benefit analysis for the routine use of HFNO in preventing hypoxia-related costs are available.^10^

## Author contributions

Conceptualisation, methodology, investigation, data curation, formal analysis, validation, writing—original draft preparation, and writing—review and editing, final approval: MC.

Methodology, investigation, data curation, formal analysis, validation, and writing—review and editing, final approval: ET, BSF, FL, ADC, PN.

Read and approved the final manuscript: all authors.

## Declarations of interest

The authors declare that they have no conflicts of interest.

## Data and materials

The datasets used and analysed during the current study are available from the corresponding author on reasonable request.
