# Supplementary material for: High-flow nasal oxygenation during gastrointestinal endoscopy. Systematic review and meta-analysis
Source: BJA Open. 2022 Oct 18;4:100098. doi: 10.1016/j.bjao.2022.100098 (PMC10430836; doi:10.1016/j.bjao.2022.100098)
Supplement: Multimedia component 2 [file mmc2.pdf]

Search strategy

**PubMed**	((oxygen inhalation therapy) AND (digestive system endoscopy)) OR (endoscopy, gastrointestinal) Filters: Full text, Controlled Clinical Trial, Randomized Controlled Trial, Humans, English, Adult: 19+ years<br>((("oxygen inhalation therapy"[MeSH Terms] OR ("oxygen"[All Fields] AND "inhalation"[All Fields] AND "therapy"[All Fields]) OR "oxygen inhalation therapy"[All Fields]) AND ("endoscopy, digestive system"[MeSH Terms] OR ("endoscopy"[All Fields] AND "digestive"[All Fields] AND "system"[All Fields]) OR "digestive system endoscopy"[All Fields] OR ("digestive"[All Fields] AND "system"[All Fields] AND "endoscopy"[All Fields]))) OR ("endoscopy, gastrointestinal"[MeSH Terms] OR ("endoscopy"[All Fields] AND "gastrointestinal"[All Fields]) OR "gastrointestinal endoscopy"[All Fields] OR ("endoscopy"[All Fields] AND "gastrointestinal"[All Fields]) OR "endoscopy gastrointestinal"[All Fields])) AND ((controlledclinicaltrial[Filter] OR randomizedcontrolledtrial[Filter]) AND (fft[Filter]) AND (humans[Filter]) AND (english[Filter]) AND (alladult[Filter]))
**Scopus**	(TITLE-ABS-KEY (oxygen AND inhalation AND therapy) AND TITLE-ABS-KEY (digestive AND system AND endoscopy) OR TITLE-ABS-KEY (endoscopy, AND gastrointestinal)) AND (LIMIT-TO (DOCTYPE , "ar")) AND (LIMIT-TO (LANGUAGE , "English"))
**Web of Science**	oxygen inhalation therapy (All Fields) AND digestive system endoscopy (All Fields) OR endoscopy, gastrointestinal (All Fields)
**Cochrane Central Register of Controlled Trials**	oxygen inhalation therapy (MeSH) AND digestive system endoscopy (MeSH) OR endoscopy, gastrointestinal (MeSH) AND NOT review AND NOT meta-analyses AND NOT case reports AND NOT cohort
