# Supplementary material for: High-flow nasal oxygenation during gastrointestinal endoscopy. Systematic review and meta-analysis
Source: BJA Open. 2022 Oct 18;4:100098. doi: 10.1016/j.bjao.2022.100098 (PMC10430836; doi:10.1016/j.bjao.2022.100098)
Supplement: Multimedia component 3 [file mmc3.pdf]

**Risk of bias assessment based on revised Cochrane risk-of-bias tool for randomized trials (RoB 2).**

**Teng WN (2019)**

| Entry | Judgment | Description |
|---|---|---|
| **Randomization process** | *Some concerns* | Randomization using a computer-generated random allocation sequence. Allocation sequence concealed not reported. No apparent imbalances. |
| **Deviations from intended interventions** | *Some concerns* | Anesthesiologist delivering the interventions aware of participants' assigned intervention. No apparent imbalances. |
| **Missing outcome data** | *Low risk* | Data about outcomes available for all participants. Result not biased by missing outcome data. No apparent imbalances. |
| **Measurement of outcome** | *Low risk* | Measuring outcomes appropriated. Outcome assessor not aware of the intervention received by study participants. No apparent imbalances. |
| **Selection of reported result** | *Low risk* | Analysis intentions available. |
| **Overall RoB2** | *Some concerns* | |

**Lin Y (2019)**

| Entry | Judgment | Description |
|---|---|---|
| **Randomization process** | *Low risk* | Randomization using a computer-generated random allocation sequence. Allocation sequence concealed reported. No apparent imbalances. |
| **Deviations from intended interventions** | *Low risk* | Anesthesiologist delivering the interventions not aware of participants' assigned intervention. No apparent imbalances. |
| **Missing outcome data** | *Low risk* | Data about outcomes available for all participants. Result not biased by missing outcome data. No apparent imbalances. |
| **Measurement of outcome** | *Some concerns* | Measuring outcomes appropriated. Outcome assessor probably aware of the intervention received by study participants. No apparent imbalances. |
| **Selection of reported result** | *Low risk* | Analysis intentions available. |
| **Overall RoB2** | *Some concerns* | |

**Riccio CA (2019)**

| Entry | Judgment | Description |
|---|---|---|
| **Randomization process** | *Low risk* | Randomization using a computer-generated random allocation sequence. Allocation sequence concealed reported. No apparent imbalances. |
| **Deviations from intended interventions** | *Some concerns* | Anesthesiologist delivering the interventions aware of participants' assigned intervention. No apparent imbalances. |
| **Missing outcome data** | *Low risk* | Data about outcomes available for all participants. Result not biased by missing outcome data. No apparent imbalances. |
| **Measurement of outcome** | *Some concerns* | Measuring outcomes appropriated. Outcome assessor probably aware of the intervention received by study participants. No apparent imbalances. |
| **Selection of reported result** | *Low risk* | Analysis intentions available. |
| **Overall RoB2** | *Some concerns* | |

**Nay MA (2021)**

| Entry | Judgment | Description |
|---|---|---|
| **Randomization process** | *Low risk* | Randomization using a computer-generated random allocation sequence. Allocation sequence concealed reported. No apparent imbalances. |
| **Deviations from intended interventions** | *Some concerns* | Anesthesiologist delivering the interventions aware of participants' assigned intervention. No apparent imbalances. |
| **Missing outcome data** | *Low risk* | Data about outcomes available for all participants. Result not biased by missing outcome data. No apparent imbalances. |
| **Measurement of outcome** | *Low risk* | Measuring outcomes appropriated. Outcome assessor not aware of the intervention received by study participants. No apparent imbalances. |
| **Selection of reported result** | *Low risk* | Analysis intentions available. |
| **Overall RoB2** | *Some concerns* | |

**Kim SH (2021)**

| Entry | Judgment | Description |
|---|---|---|
| **Randomization process** | *Some concerns* | Randomization using a computer-generated random allocation sequence. Allocation sequence concealed not reported. No apparent imbalances. |
| **Deviations from intended interventions** | *Some concerns* | Anesthesiologist delivering the interventions aware of participants' assigned intervention. No apparent imbalances. |
| **Missing outcome data** | *Low risk* | Data about outcomes available for all participants. Result not biased by missing outcome data. No apparent imbalances. |
| **Measurement of outcome** | *Some concerns* | Measuring outcomes appropriated. Outcome assessor probably aware of the intervention received by study participants. No apparent imbalances. |
| **Selection of reported result** | *Low risk* | Analysis intentions available. |
| **Overall RoB2** | *Some concerns* | |

**Mazzeffi MA (2021)**

| Entry | Judgment | Description |
|---|---|---|
| **Randomization process** | *Low risk* | Randomization using a computer-generated random allocation sequence. Allocation sequence concealed reported. No apparent imbalances. |
| **Deviations from intended interventions** | *Some concerns* | Anesthesiologist delivering the interventions aware of participants' assigned intervention. No apparent imbalances. |
| **Missing outcome data** | *Low risk* | Data about outcomes available for all participants. Result not biased by missing outcome data. No apparent imbalances. |
| **Measurement of outcome** | *Low risk* | Measuring outcomes appropriated. Outcome assessor not aware of the intervention received by study participants. No apparent imbalances. |
| **Selection of reported result** | *Low risk* | Analysis intentions available. |
| **Overall RoB2** | *Some concerns* | |
