# Supplementary material for: High-flow nasal oxygenation during gastrointestinal endoscopy. Systematic review and meta-analysis
Source: BJA Open. 2022 Oct 18;4:100098. doi: 10.1016/j.bjao.2022.100098 (PMC10430836; doi:10.1016/j.bjao.2022.100098)
Supplement: Multimedia component 4 [file mmc4.pdf]

SUPPLEMENTARY MATERIAL CONTENT (SMC) 5 GRADE (GRADES OF RECOMMENDATION, ASSESSMENT, DEVELOPMENT, AND EVALUATION)

Author(s): Carron - Tamburini - Safaee Fakhr - Linassi - De Cassai - Navalesi

Question: High-flow nasal oxygen compared to conventional oxygen therapy for reducing incidence of adverse events during sedation for endoscopic procedure

Setting: Anesthesia

Bibliography:

1. *Randomized controlled trials*: Lin Y et al. Gastrointest Endosc 2019 - Mazzeffi et al. Anesth Analg 2021 - Teng et al. Biomed Res Int. 2019 - Riccio et al. J Clin Anesth. 2019 - Nay et al. Br J Anaesth 2021 - Kim et al. Can J Anaesth 2021
2. *No randomized controlled trials*: Agostoni M et al. Gastrointest Endosc. 2011 - Goudra B et al. Saudi J Gastroenterol. 2015 - Goudra B et al. Clin Endosc. 2017 - Agostoni M et al Gastrointest Endosc. 2011

Certainty assessment							№ of patients		Effect		Certainty	Importance
№ of studies	Study design	Risk of bias	Inconsistency	Indirectness	Imprecision	Other considerations	High-flow nasal oxygen	conventional oxygen therapy	Relative (95% CI)	Absolute (95% CI)		
**Respiratory hypoxic events during sedation for gastrointestinal endoscopy**												
6	randomised trials	not serious[a]	serious[b]	not serious	not serious	strong association[c]	58/1431 (4.1%)	222/1436 (15.5%)	**RR 0.23** (0.07 to 0.70)	**119 fewer per 1.000** (from 144 fewer to 46 fewer)	⨁⨁⨁⨁ High	IMPORTANT[d]

CI: confidence interval; **RR:** risk ratio

Explanations

a. Anesthesiologists delivering the interventions were aware of participants' assigned intervention. No apparent imbalances were noted.

b. A substantial heterogeneity (I^2 >50%) was observed.

c. A large effect was observed (RR >2 or <0.5).

d. This event increases the risk of adverse outcomes (please see bibliography).
