# Supplementary material for: High-flow nasal oxygenation during gastrointestinal endoscopy. Systematic review and meta-analysis
Source: BJA Open. 2022 Oct 18;4:100098. doi: 10.1016/j.bjao.2022.100098 (PMC10430836; doi:10.1016/j.bjao.2022.100098)
Supplement: Multimedia component 5 [file mmc5.pdf]

General population of patients	RR	95%CI	P-value	I^2
Hypoxic events	0.2374	[0.0794; 0.7092]	0.0100	93.8%
-hypoxic events (SpO_2<90%)	0.1289	[0.0233; 0.7146]	0.0190	90.4%
Hypoxic events with HFNO ≥40 $L \cdot min^{-1}$	0.2103	[0.0577; 0.7671]	0.0182	93.4%
Hypoxic events with HFNO <40 $L \cdot min^{-1}$	0.3164	[0.0510; 1.9628]	0.2166	70.7%
Hypoxic events in short procedure	0.0650	[0.0401; 0.1054]	<0.0001	0.0%
-hypoxic events in short procedure (SpO_2<90%)	0.0256	[0.0010; 0.6710]	0.0278	72.6%
Hypoxic events in long procedure	0.4820	[0.2469; 0.9409]	0.0325	76.6%
-hypoxic events in long procedure (SpO_2<90%)	0.3592	[0.1043; 1.2370]	0.1046	81.7%
Rescue treatment	0.1905	[0.0401; 0.9054]	0.0371	96.2%
-minor rescue treatment	0.1326	[0.0183; 0.9588]	0.0453	97.4%
-major rescue treatment	0.5687	[0.2555; 1.2659]	0.1669	0.0%
Procedure interruption	0.1167	[0.0286; 2.2926]	0.0132	0.0%
Total adverse events	0.8785	[0.5527; 1.3963]	0.5838	94.6%
-respiratory no-hypoxaemic events	1.0538	[0.7824; 1.4191]	0.7303	56.2%
-cardiovascular events	0.9041	[0.6307; 1.2960]	0.5832	43.6%
Hypoxic events with sedation	0.0730	[0.0000; 1299.6660]	0.6002	97.9%
Hypoxic events with sedation and opioid	0.0830	[0.0161; 0.4269]	0.0029	0.0%
**Non-obese patients**				
Hypoxic events	0.1134	[0.0392; 0.3277]	0.0001	76.3%
-hypoxic events (SpO2<90%)	0.0614	[0.0106; 0.3558]	0.0019	66.6%
Rescue treatment	0.0294	[0.0143; 0.0602]	< 0.0001	4.7%
-minor rescue treatment	0.0248	[0.0126; 0.0487]	< 0.0001	0.0%
-major rescue treatment	0.2562	[0.0286; 2.2926]	0.2232	0.0%
Total adverse events	0.8001	[0.2896; 2.2107]	0.6671	96.8%
-respiratory no-hypoxaemic events	0.6616	[0.1307; 3.3486]	0.6176	84.3%
-cardiovascular events	0.7779	[0.5125; 1.1808]	0.2382	0.0%
**Obese Patients**				
Hypoxic events	0.5565	[0.2188; 1.4152]	0.2184	75.3%
-hypoxic events (SpO_2<90%)	0.6101	[0.2770; 1.3436]	0.2199	61.2%

Hypoxic events were defined as "desaturation" (decreased peripheral capillary oxygen saturation [SpO_2]) according with the studies' endpoints observed after the induction and the maintenance of sedation for gastrointestinal endoscopy. A subgroup analysis evaluated hypoxic events defined as SpO_2<90%. General population included both non-obese and obese patients. Obesity: Body Mass Index (BMI) of ≥30 $kg \cdot m^{-2}$. RR: relative risk; 95%CI: 95% confidence interval.
