# Supplementary material for: High-flow nasal oxygenation during gastrointestinal endoscopy. Systematic review and meta-analysis
Source: BJA Open. 2022 Oct 18;4:100098. doi: 10.1016/j.bjao.2022.100098 (PMC10430836; doi:10.1016/j.bjao.2022.100098)

Statistical analysis

The meta-analysis was performed within a frequentist framework using both random and fixed effects models, computing the relative risk (RR) and 95% confidence interval (CI) for binary outcome data. The Mantel-Haenszel method was used to calculate the fixed effects estimate for dichotomous data. When calculating the RR, 0.5 was added to the frequencies of all studies with a zero number of events. The random effects model was computed with inverse-variance weighting using the DerSimonian and Laird method to account for heterogeneity. Heterogeneity across studies was tested using the I^2 statistic. A threshold of $P<0.1$ was used to determine whether heterogeneity was present. I^2 was considered low (< 25%), moderate (25-50%), or high (>50%). The random effects model was preferred for final analysis. If the number of included studies was very small, then it would be impossible to estimate the between-studies variance (tau-squared) with any precision. In this event, it will be considered the results of the Q test to identify substantial heterogeneity.

Hypoxic events were defined as "desaturation" (decreased peripheral capillary oxygen saturation [SpO_2]) according with the studies' endpoints observed after the induction and the maintenance of sedation for gastrointestinal endoscopy. A subgroup analysis evaluated hypoxic events defined as SpO_2<90%.

General population included both non-obese and obese patients (Body Mass Index of ≥30 kg/m2).

Forest plots of different meta-analyses.

A forest plot of metanalysis for each outcome is provided with effect sizes and confidence intervals (CIs) for each individual study. Square's area is proportional to study weights (inverse-variance weight). The diamond-shaped polygon at the bottom shows the model summary estimate, with the centre of the polygon corresponding to the estimate and the left/right edges indicating the confidence interval limits.

General population of patients

Hypoxic events

Author(s); Year / Outcome	Effect size [95%CI]
Teng_2019	-2.38 [-4.39, -0.37]
Lin_2019	-2.75 [-3.25, -2.26]
Riccio_2019	-0.14 [-0.74, 0.46]
Nay_2021	-1.27 [-1.75, -0.79]
Kim_2021	-2.71 [-5.53, 0.12]
Mazzeffi_2021	-0.44 [-0.85, -0.03]
Estimated effect	-1.37 [-2.20, -0.54]

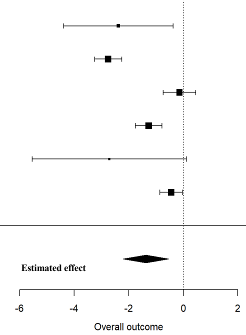

Hypoxic events - HFNO ≥40 $L \cdot min^{-1}$

Author(s); Year / Outcome	Effect size [95%CI]
Lin_2019	-2.75 [-3.25, -2.26]
Riccio_2019	-0.14 [-0.74, 0.46]
Nay_2021	-1.27 [-1.75, -0.79]
Kim_2021	-2.71 [-5.53, 0.12]
Estimated effect	-1.54 [-2.65, -0.42]

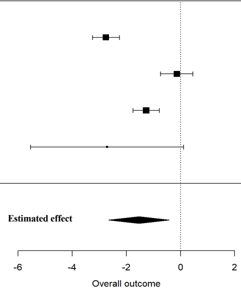

Hypoxic events – short procedure

Author(s); Year / Outcome	Effect size [95%CI]
Teng 2019	-2.38 [-4.39, -0.37]
Lin 2019	-2.75 [-3.25, -2.26]
Estimated effect	-2.73 [-3.22, -2.25]

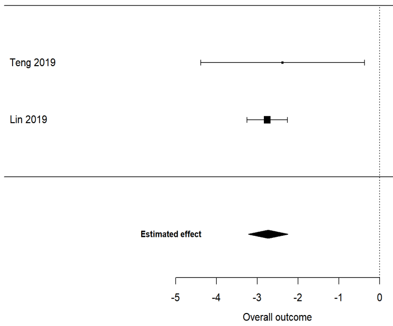

Hypoxic events - long procedure

Author(s); Year / Outcome	Effect size [95%CI]
Riccio_2019	-0.14 [-0.74, 0.46]
Nay_2021	-1.27 [-1.75, -0.79]
Kim_2021	-2.71 [-5.53, 0.12]
Mazzeffi_2021	-0.44 [-0.85, -0.03]
Estimated effect	-0.81 [-1.78, 0.17]

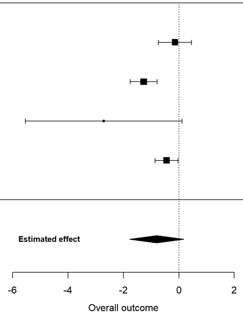

Rescue treatment

Author(s); Year / Outcome	Effect size [95%CI]
Teng_2019	-2.18 [-4.21, -0.15]
Lin_2019	-3.68 [-4.38, -2.98]
Riccio_2019	0.04 [-0.45, 0.52]
Mazzeffi_2020	0.27 [-1.20, 1.75]
Nay_2021	-1.10 [-1.40, -0.79]
Kim_2021	-4.11 [-6.87, -1.35]
Estimated effect	-1.64 [-3.10, -0.19]

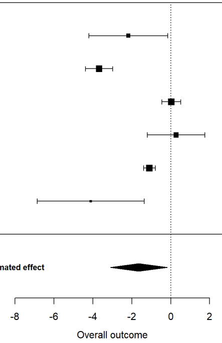

Hypoxic events SpO_2 <90%

Author(s); Year / Outcome	Effect size [95%CI]
Teng_2019	-2.38 [-4.39, -0.37]
Lin_2019	-5.19 [-7.97, -2.41]
Riccio_2019	-0.14 [-0.74, 0.46]
Nay_2021	-1.38 [-2.01, -0.75]
Kim_2021	-2.71 [-5.53, 0.12]
Estimated effect	-2.00 [-3.59, -0.41]

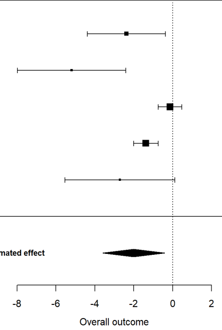

Hypoxic events - HFNO <40 $L \cdot min^{-1}$

Author(s); Year / Outcome	Effect size [95%CI]
Teng_2019	-2.38 [-4.39, -0.37]
Mazzeffi_2021	-0.44 [-0.85, -0.03]
Estimated effect	-1.15 [-2.98, 0.67]

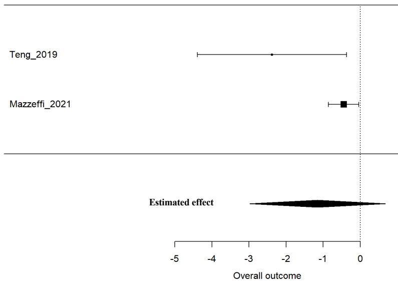

Hypoxic events SpO_2 <90% - short procedure

Author(s); Year / Outcome	Effect size [95%CI]
Teng 2019	-2.38 [-4.39, -0.37]
Lin 2019	-5.19 [-7.97, -2.41]
Estimated effect	-3.62 [-6.35, -0.88]

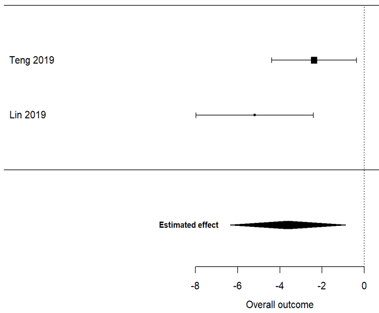

Hypoxic events SpO_2 <90% - long procedure

Author(s); Year / Outcome	Effect size [95%CI]
Riccio_2019	-0.14 [-0.74, 0.46]
Nay_2021	-1.38 [-2.01, -0.75]
Kim_2021	-2.71 [-5.53, 0.12]
Estimated effect	-1.04 [-2.31, 0.24]

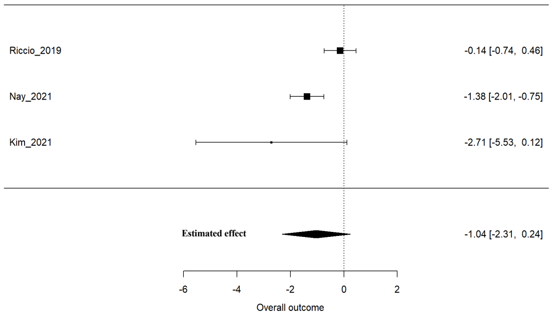

Minor rescue treatment

Author(s); Year / Outcome	Effect size [95%CI]
Lin_2019	-3.67 [-4.37, -2.98]
Riccio_2019	0.03 [-0.53, 0.58]
Nay_2021	-1.09 [-1.41, -0.77]
Kim_2021	-4.08 [-6.83, -1.32]
Estimated effect	-2.01 [-3.93, -0.09]

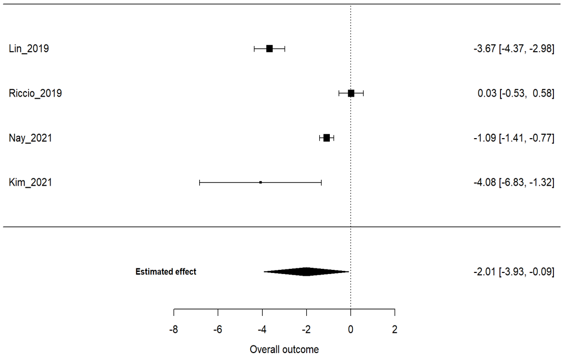

SUPPLEMENTARY MATERIAL CONTENT (SMC) 4 — Forest plots

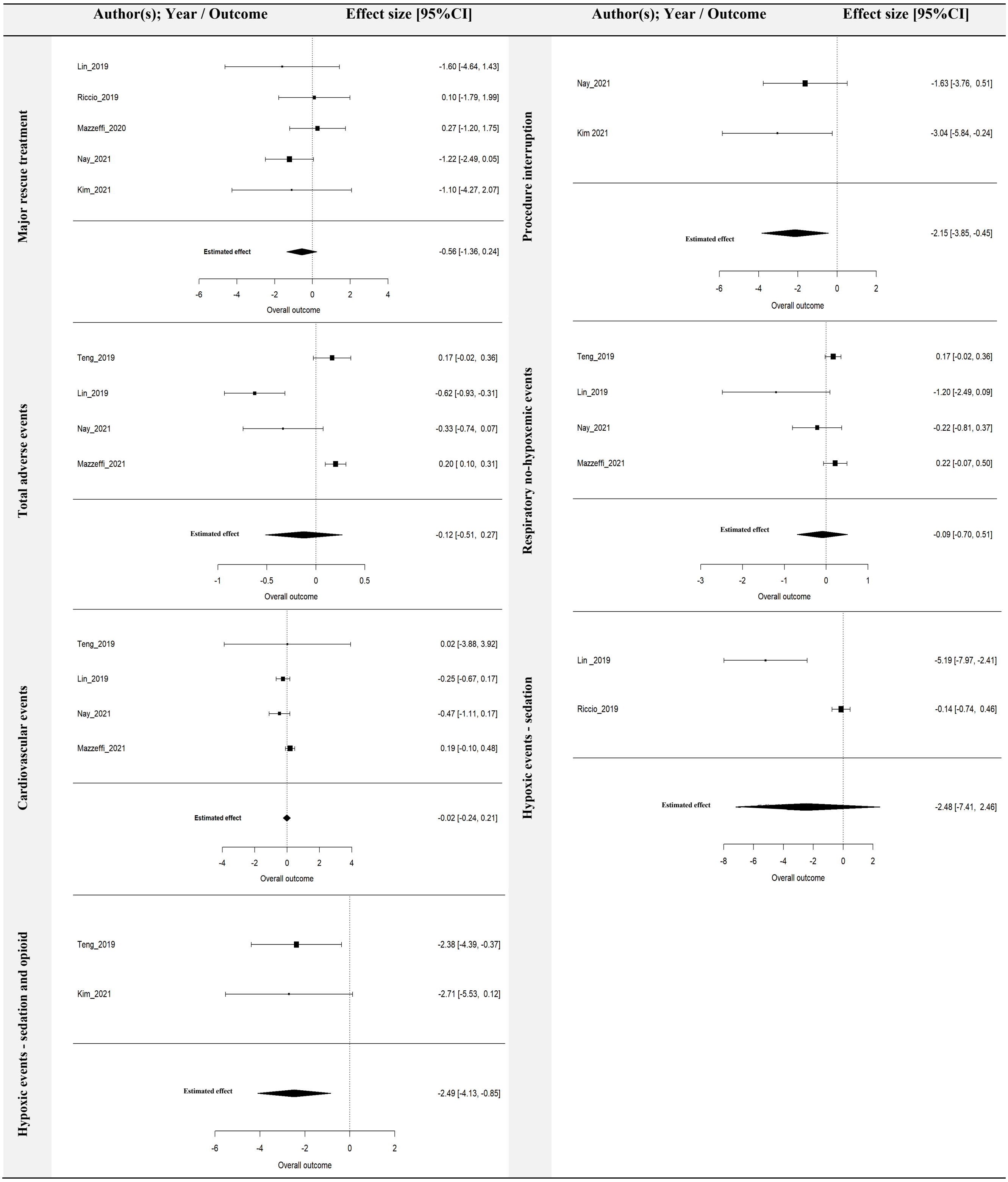

Outcome	Author(s); Year	Effect size [95%CI]
Major rescue treatment	Lin_2019	-1.60 [-4.64, 1.43]
	Riccio_2019	0.10 [-1.79, 1.99]
	Mazzeffi_2020	0.27 [-1.20, 1.75]
	Nay_2021	-1.22 [-2.49, 0.05]
	Kim_2021	-1.10 [-4.27, 2.07]
	Estimated effect	-0.56 [-1.36, 0.24]
Total adverse events	Teng_2019	0.17 [-0.02, 0.36]
	Lin_2019	-0.62 [-0.93, -0.31]
	Nay_2021	-0.33 [-0.74, 0.07]
	Mazzeffi_2021	0.20 [0.10, 0.31]
	Estimated effect	-0.12 [-0.51, 0.27]
Cardiovascular events	Teng_2019	0.02 [-3.88, 3.92]
	Lin_2019	-0.25 [-0.67, 0.17]
	Nay_2021	-0.47 [-1.11, 0.17]
	Mazzeffi_2021	0.19 [-0.10, 0.48]
	Estimated effect	-0.02 [-0.24, 0.21]
Hypoxic events - sedation and opioid	Teng_2019	-2.38 [-4.39, -0.37]
	Kim_2021	-2.71 [-5.53, 0.12]
	Estimated effect	-2.49 [-4.13, -0.85]
Procedure interruption	Nay_2021	-1.63 [-3.76, 0.51]
	Kim 2021	-3.04 [-5.84, -0.24]
	Estimated effect	-2.15 [-3.85, -0.45]
Respiratory no-hypoxemic events	Teng_2019	0.17 [-0.02, 0.36]
	Lin_2019	-1.20 [-2.49, 0.09]
	Nay_2021	-0.22 [-0.81, 0.37]
	Mazzeffi_2021	0.22 [-0.07, 0.50]
	Estimated effect	-0.09 [-0.70, 0.51]
Hypoxic events - sedation	Lin _2019	-5.19 [-7.97, -2.41]
	Riccio_2019	-0.14 [-0.74, 0.46]
	Estimated effect	-2.48 [-7.41, 2.46]

SUPPLEMENTARY MATERIAL CONTENT (SMC) 4 — Forest plots

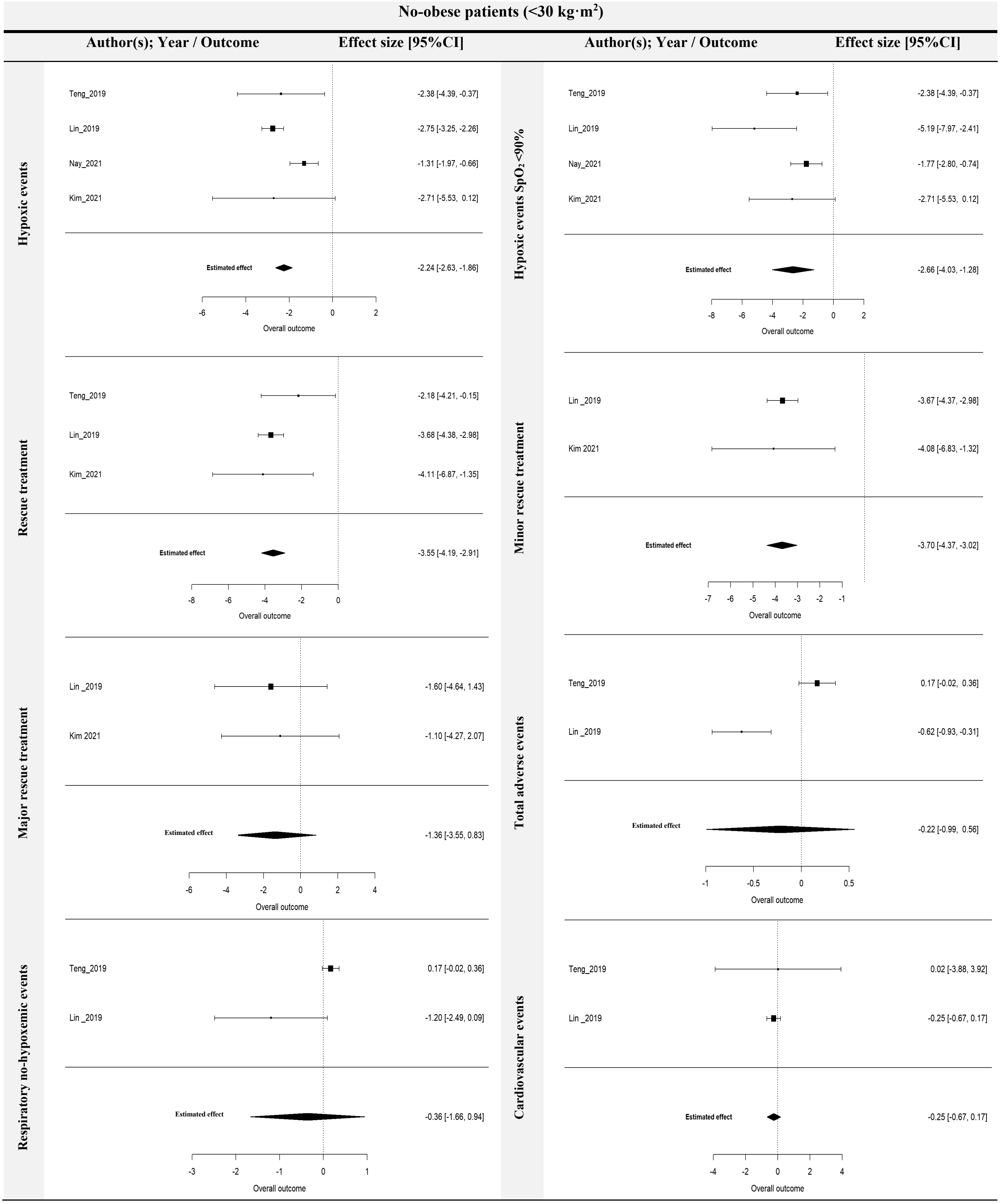

Obese patients (≥30 kg·m^2)

Author(s); Year / Outcome	Effect size [95%CI]	Author(s); Year / Outcome	Effect size [95%CI]

Hypoxic events

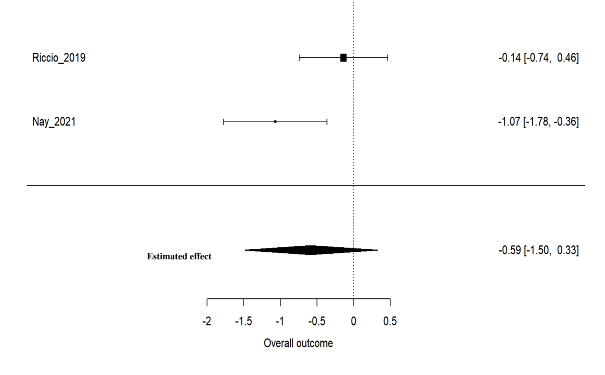

Hypoxic events SpO_2 <90%

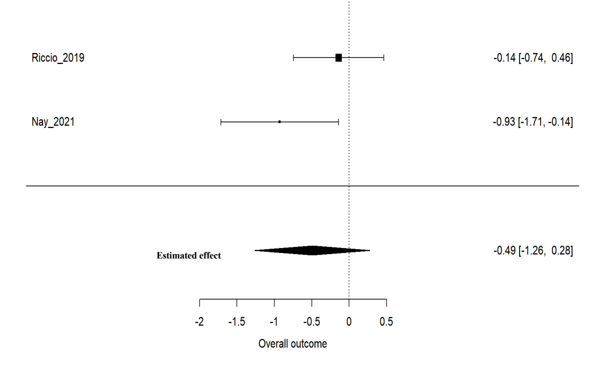

Supplement: Multimedia component 6 [file mmc6.pdf]
