# Supplementary material for: High-flow nasal oxygenation during gastrointestinal endoscopy. Systematic review and meta-analysis
Source: BJA Open. 2022 Oct 18;4:100098. doi: 10.1016/j.bjao.2022.100098 (PMC10430836; doi:10.1016/j.bjao.2022.100098)

A funnel plot of metanalysis for each outcome is provided to represent the effect estimates (Log Risk Ratio) from individual studies against standard errors. The standard error is plotted on the vertical axis with a reversed scale that places the larger, most powerful studies towards the top. The outer dashed lines indicate the triangular region within which 95% of studies are expected to lie in the absence of both biases and heterogeneity.

Outliers resulting from leave-one-out diagnostics were evaluated to establish which studies have a strong influence on the results (as reflected, for example, in their Cook's distances). Rstudent: studentized residuals; cooks.distance: Cook’s distance; Cov.r: covariance ratio; Tau2.del: leave-one-out estimate of heterogeneity; QE.del: chi-square test for homogeneity; Hat: hat value; Weight: weight of a study in the overall meta-analytic average effect size.

Forrest plots meta-analyses and outliers resulting from leave-one out diagnostics

General population of patients

Hypoxic events

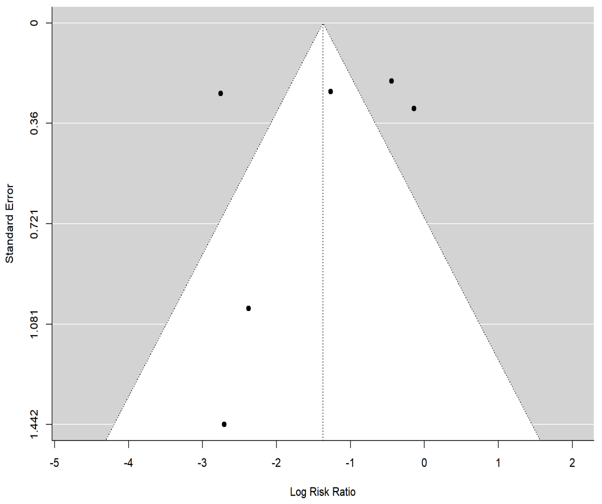

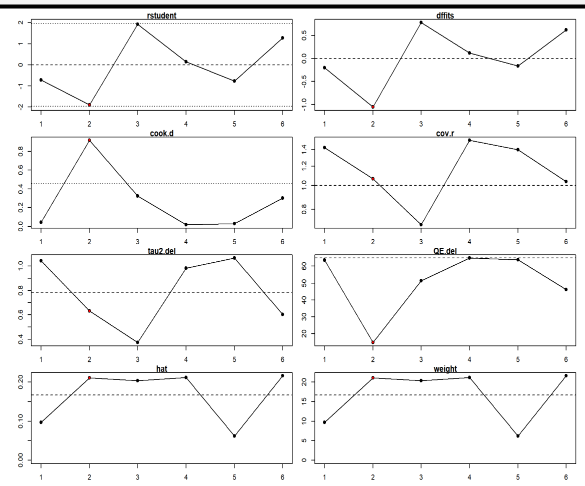

Hypoxic events SpO_2<90%

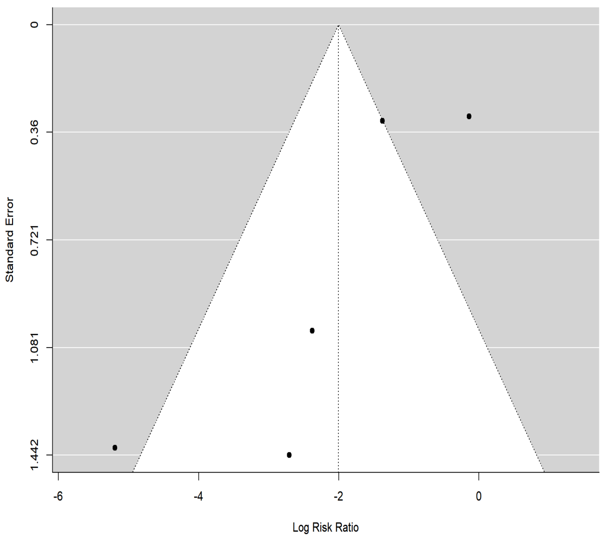

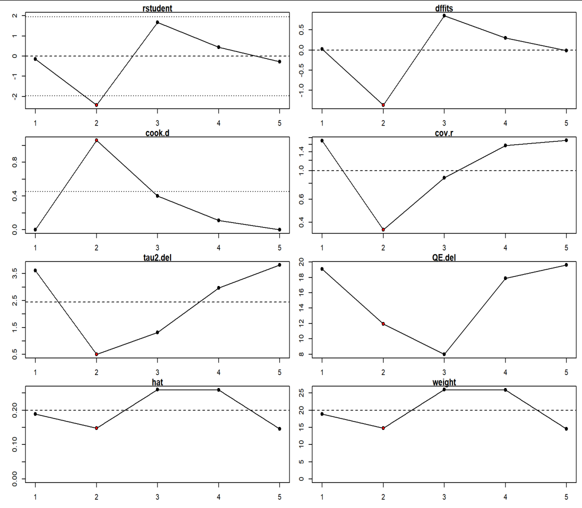

Hypoxic events with HFNO ≥40 L·min^{-1}

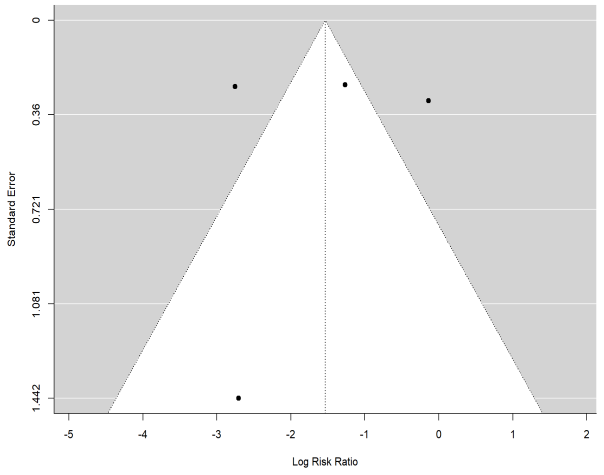

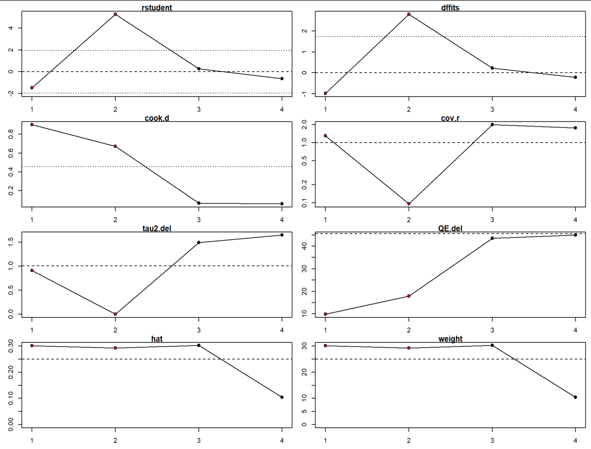

Hypoxic events with HFNO <40 L·min^{-1}

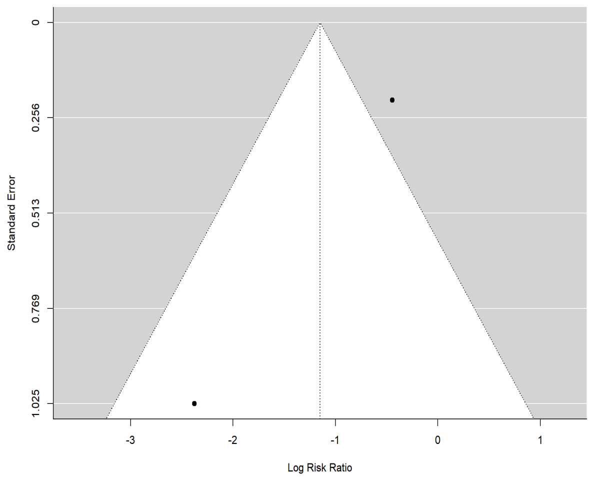

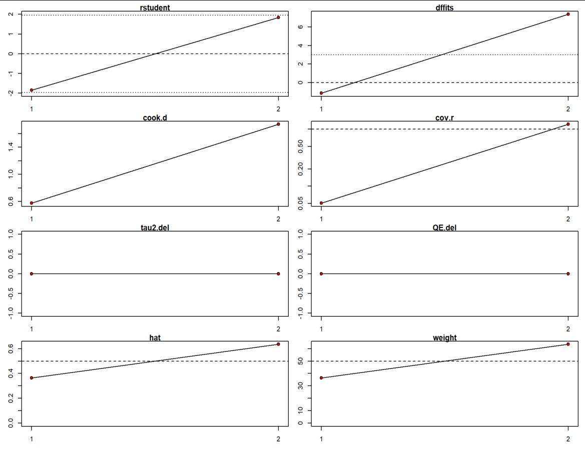

Hypoxic events in short procedure

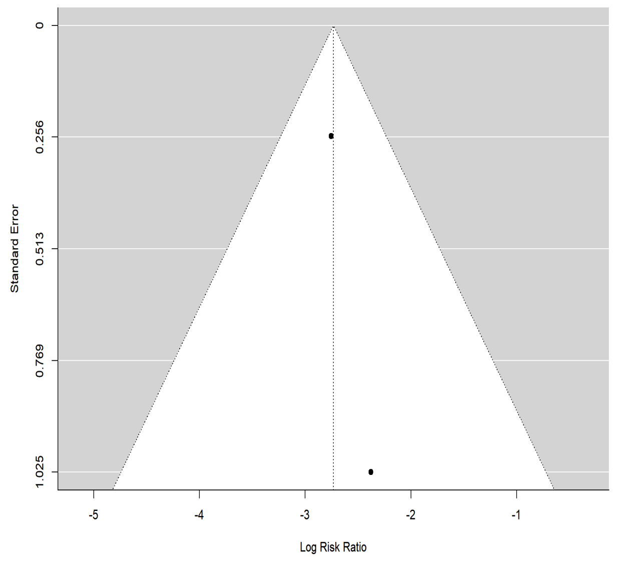

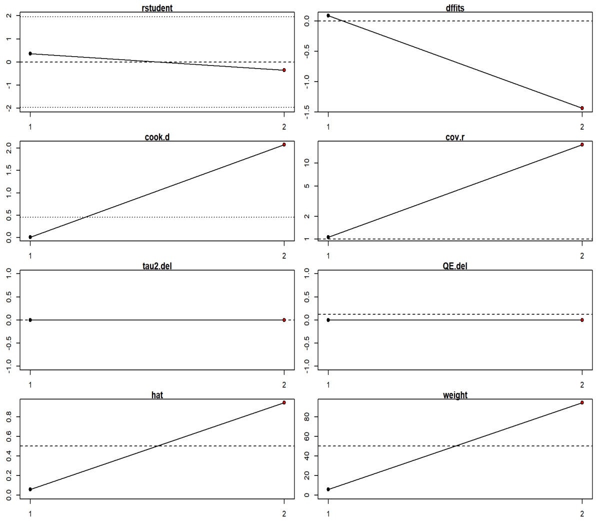

Hypoxic events SpO_2<90% in short procedure

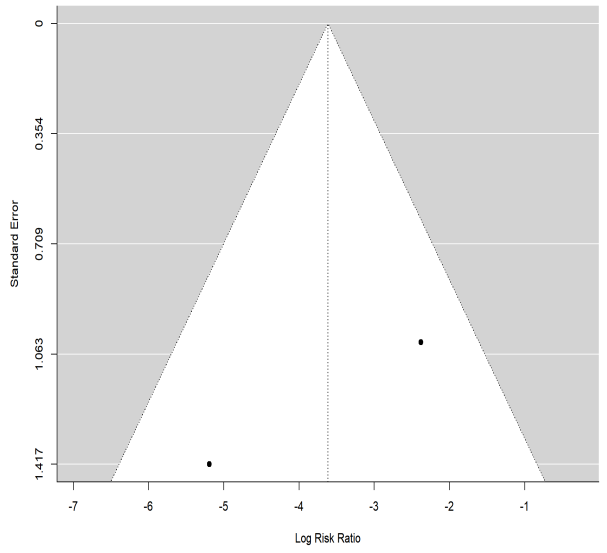

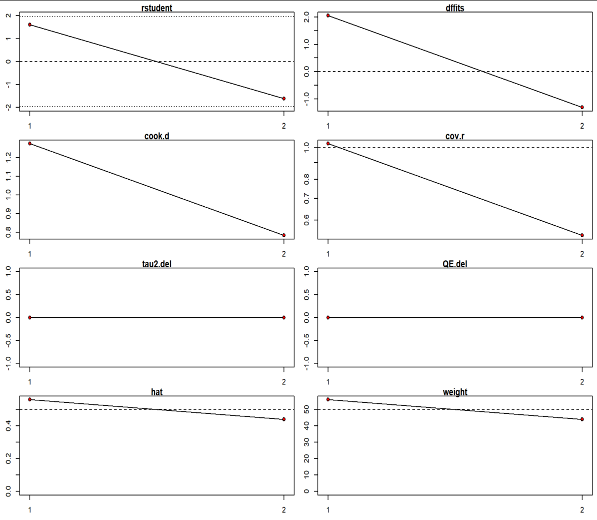

Hypoxic events in long procedure

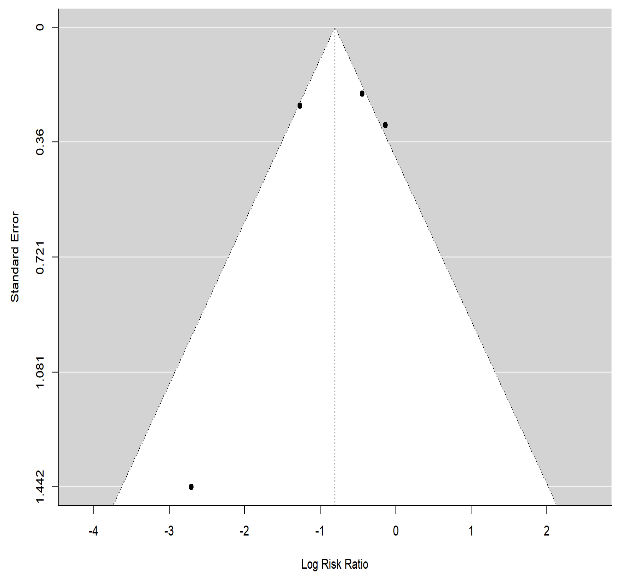

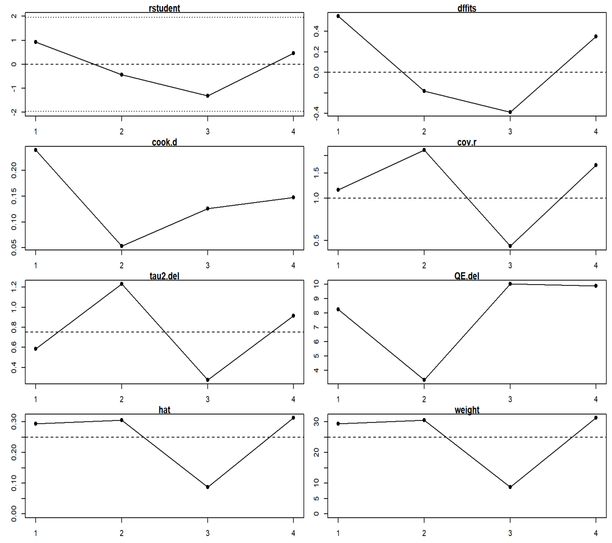

Hypoxic events SpO_2<90% in long procedure

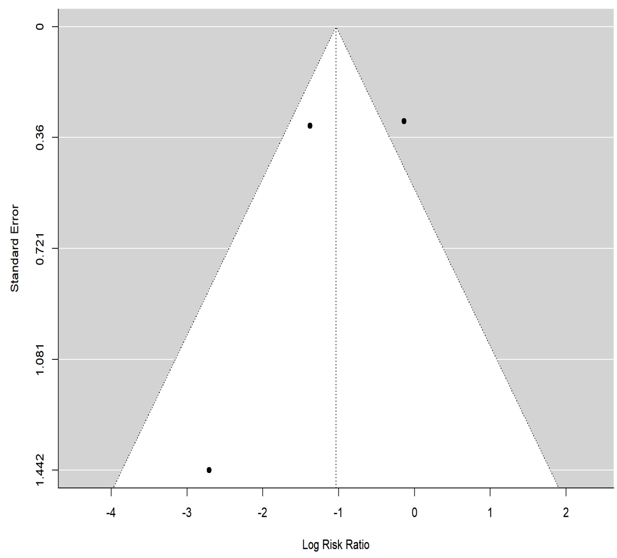

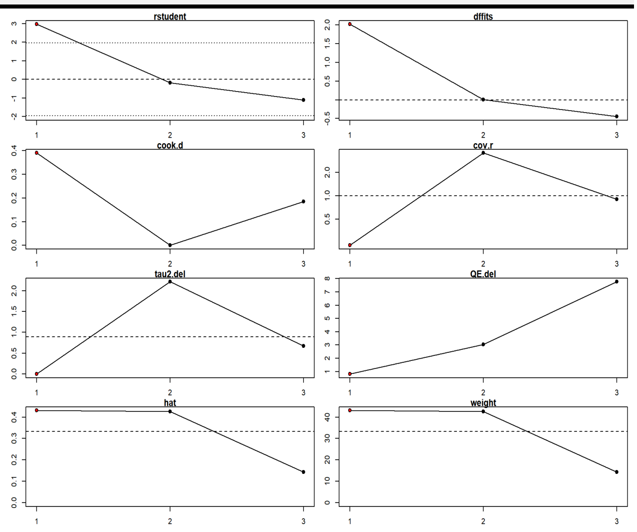

Rescue treatment

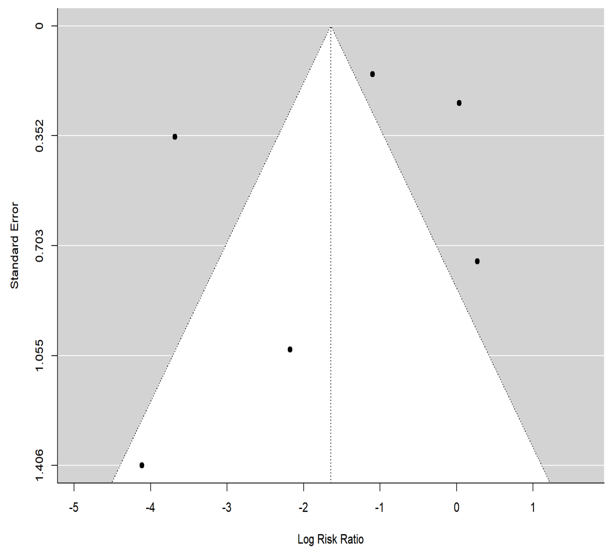

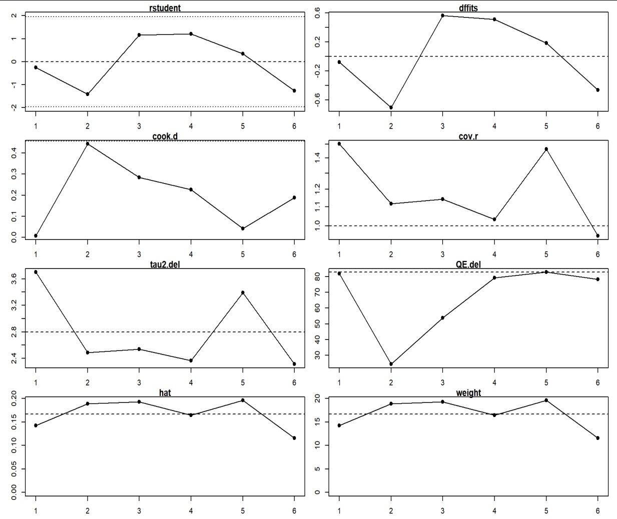

Minor rescue treatment

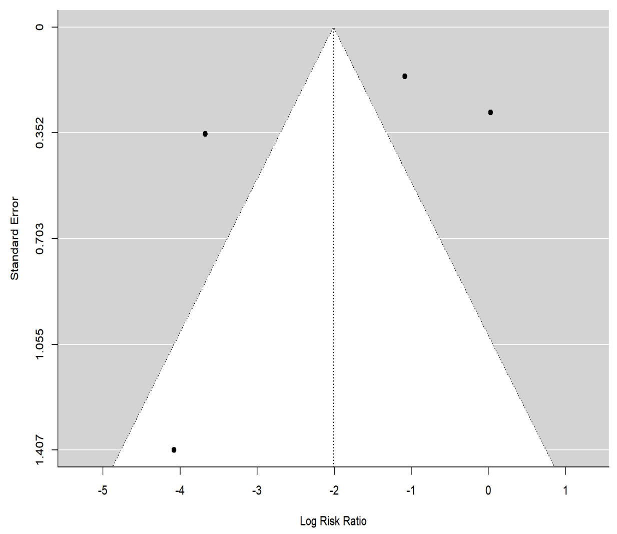

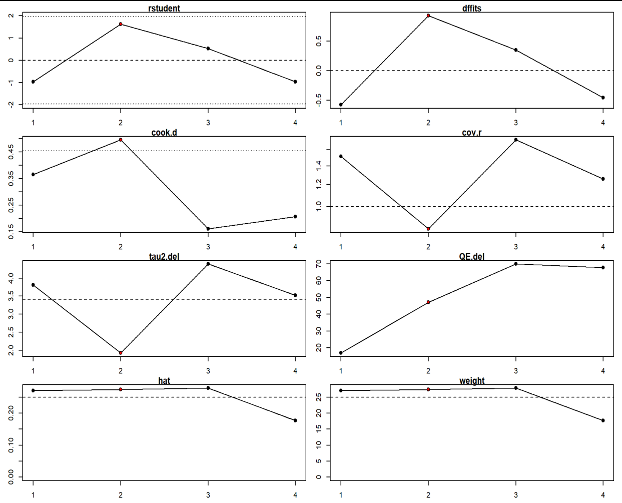

Major rescue treatment

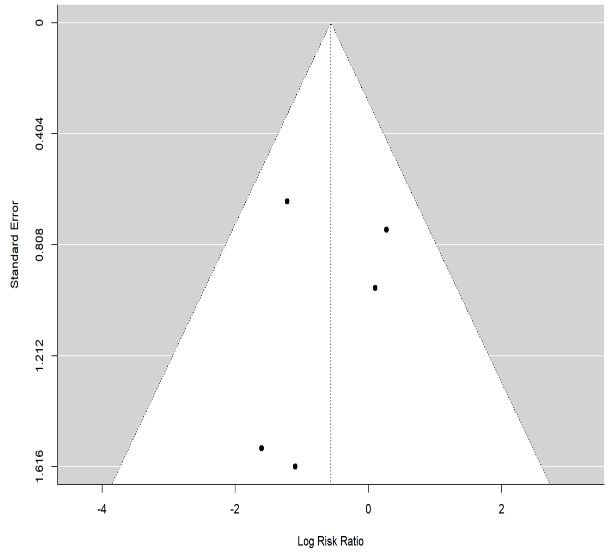

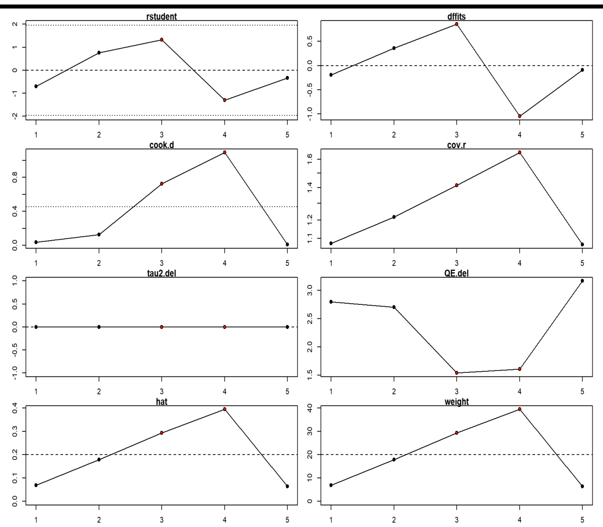

Procedure interruption

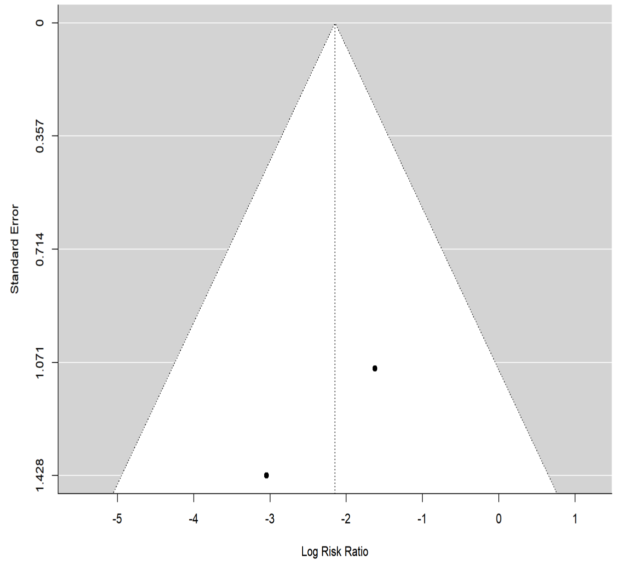

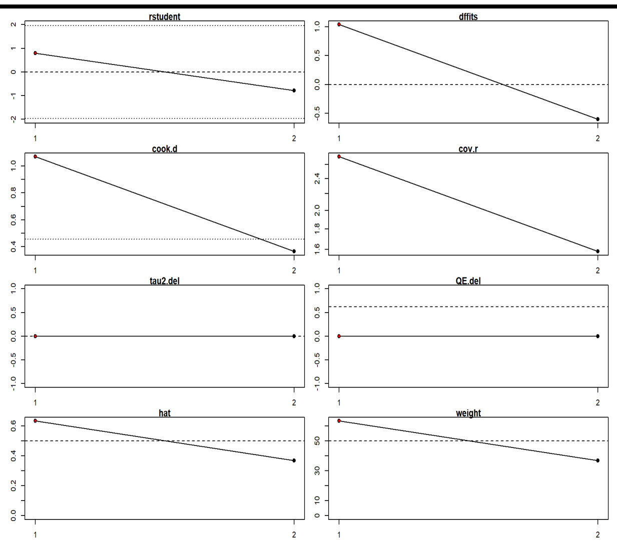

Total adverse events

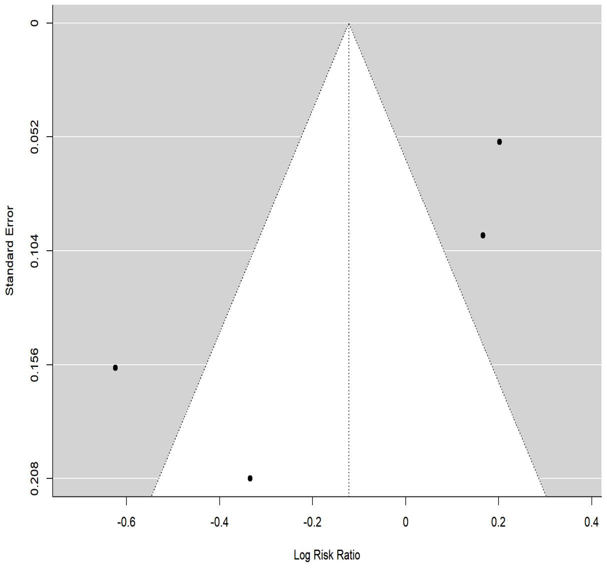

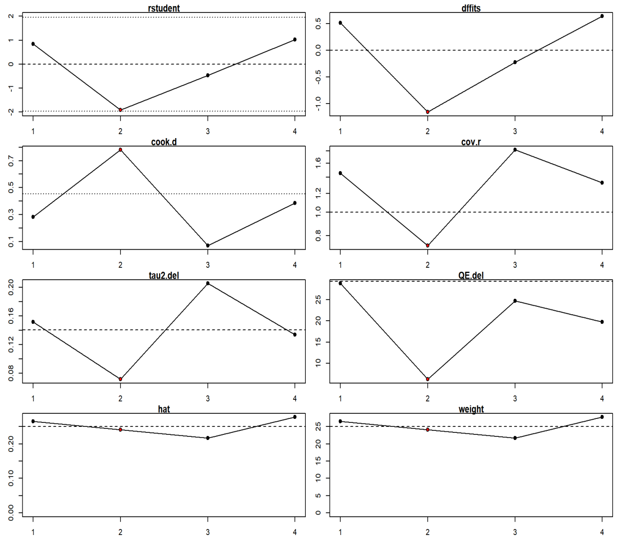

Respiratory no-hypoxemic events

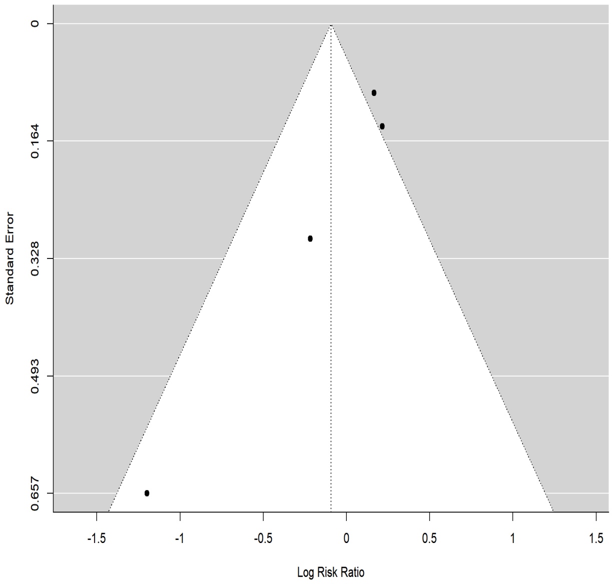

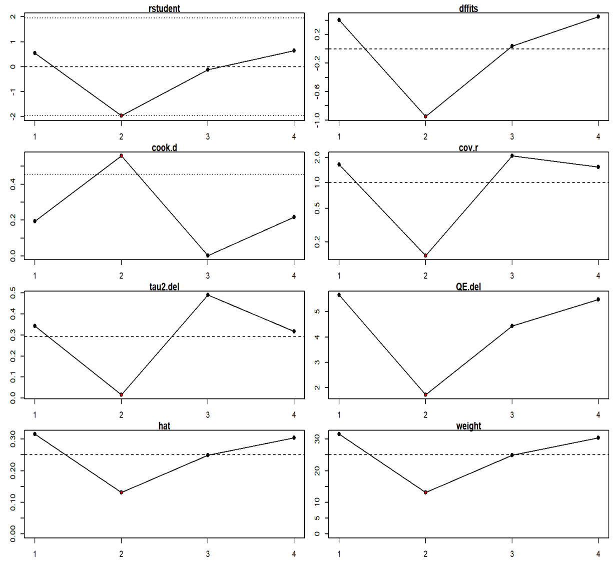

Cardiovascular events

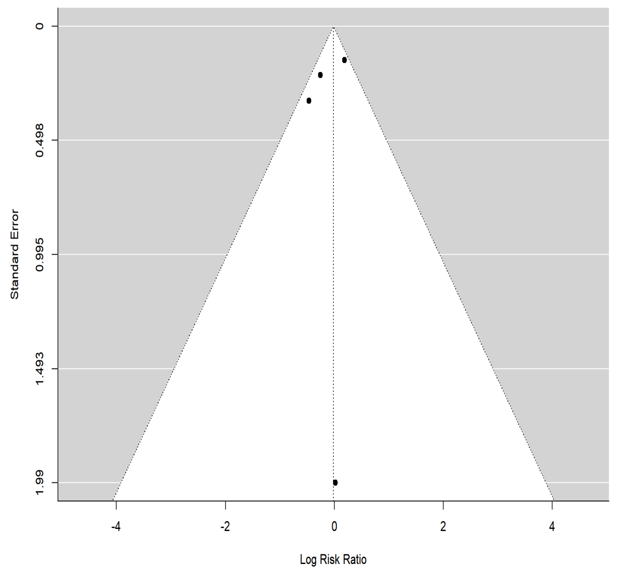

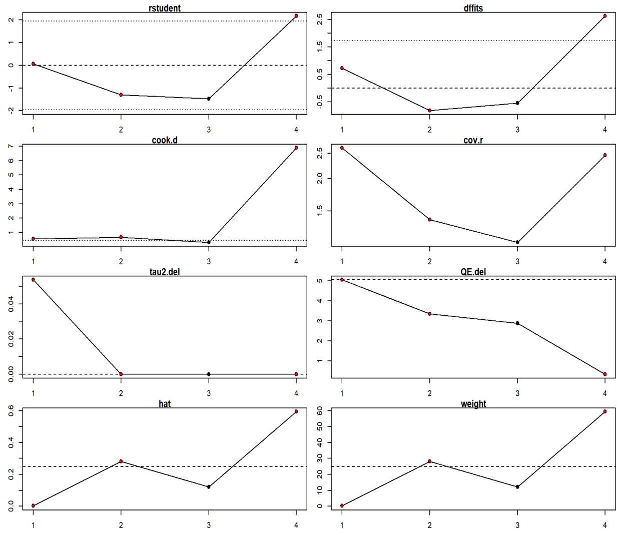

Hypoxic events with sedation

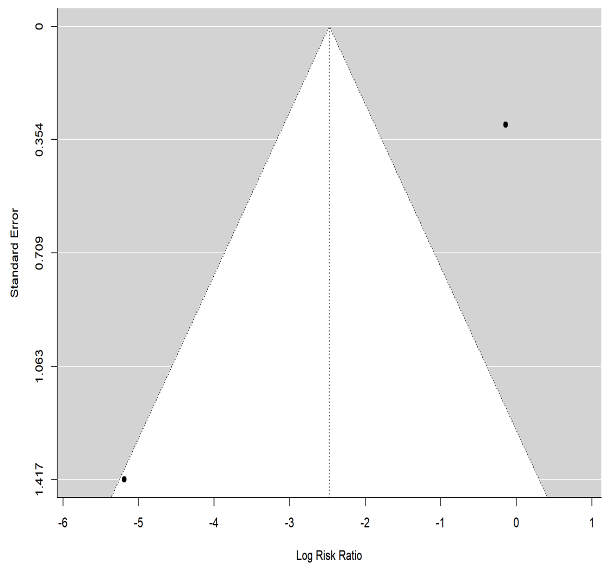

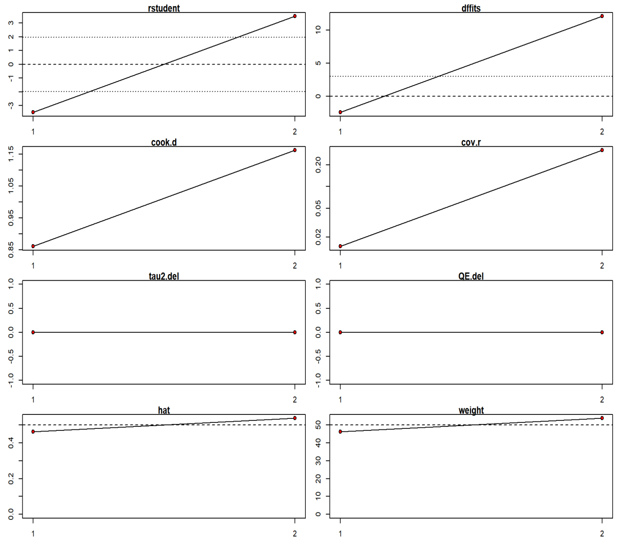

Hypoxic events with sedation and opioid

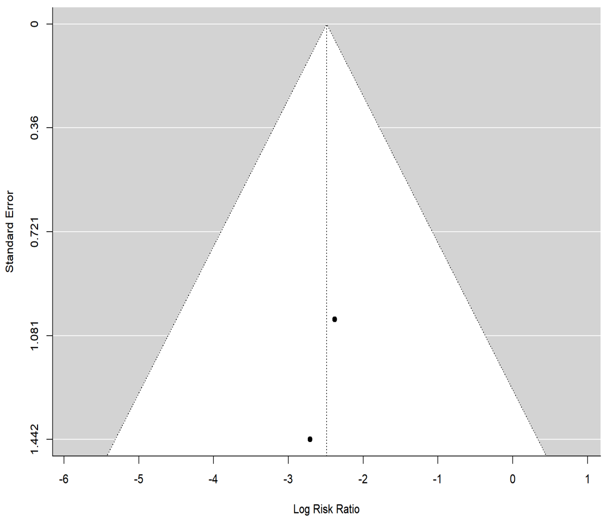

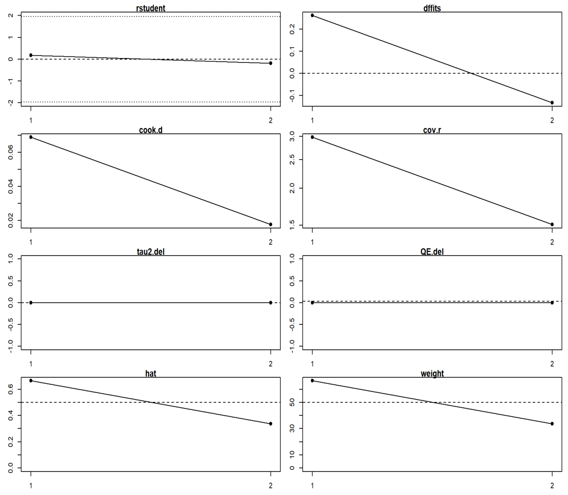

No-obese patients (<30 kg/m^2)

Hypoxic events

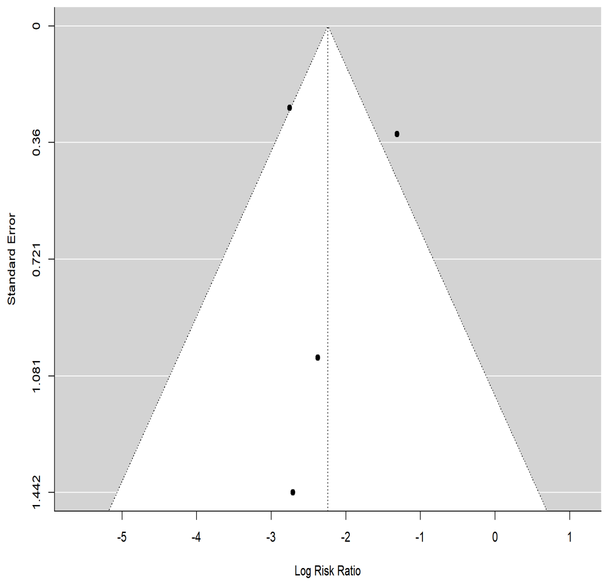

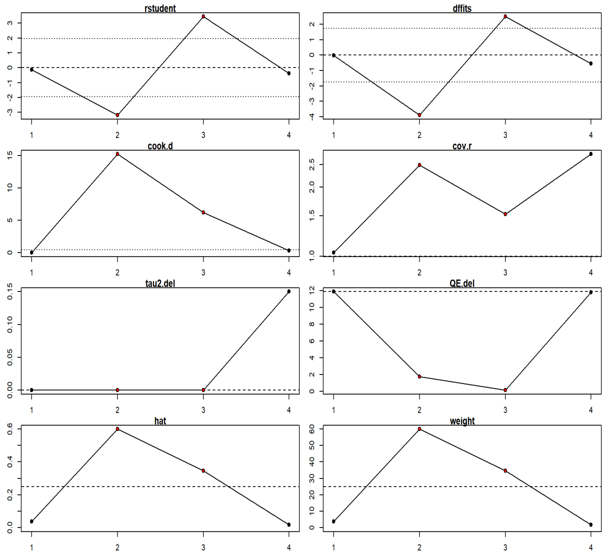

Hypoxic events SpO_2<90%

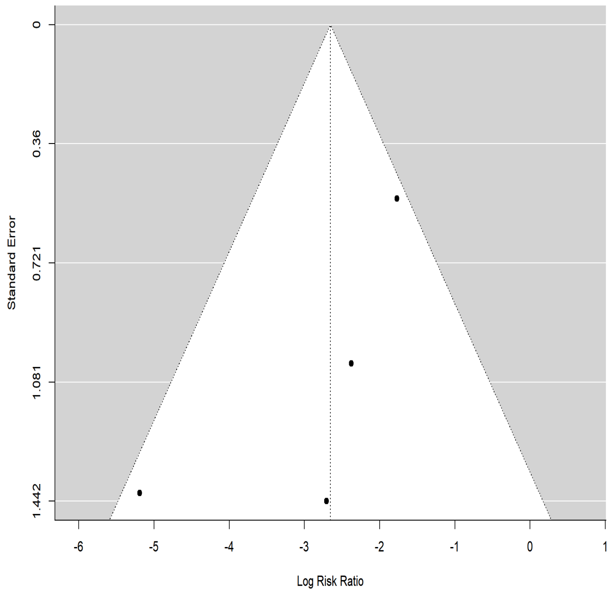

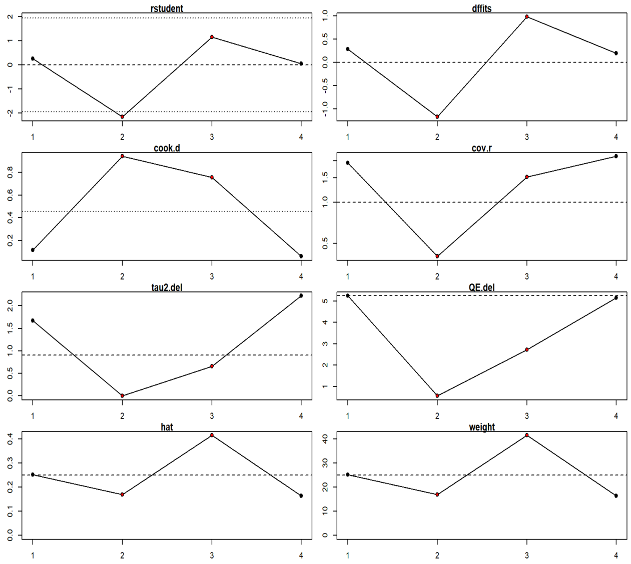

Rescue treatment

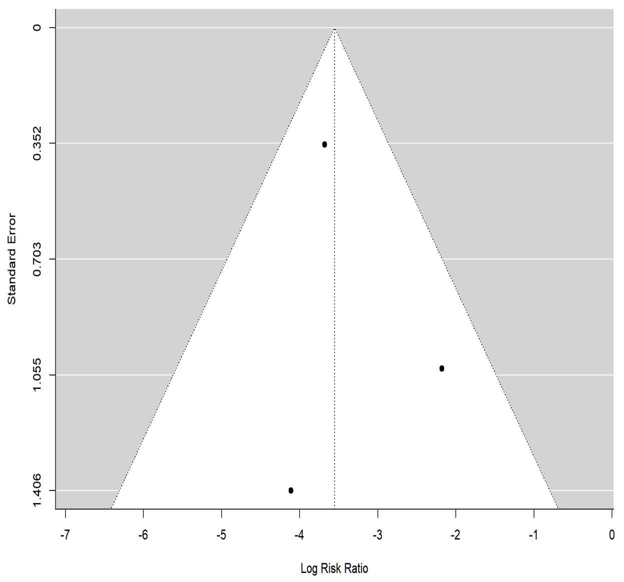

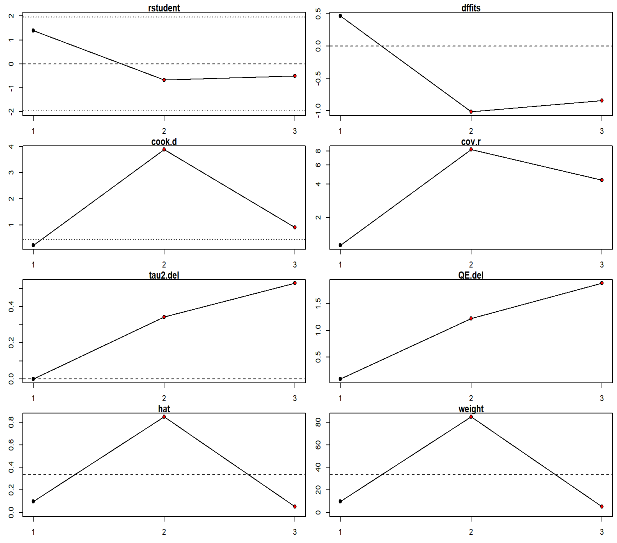

Minor rescue treatment

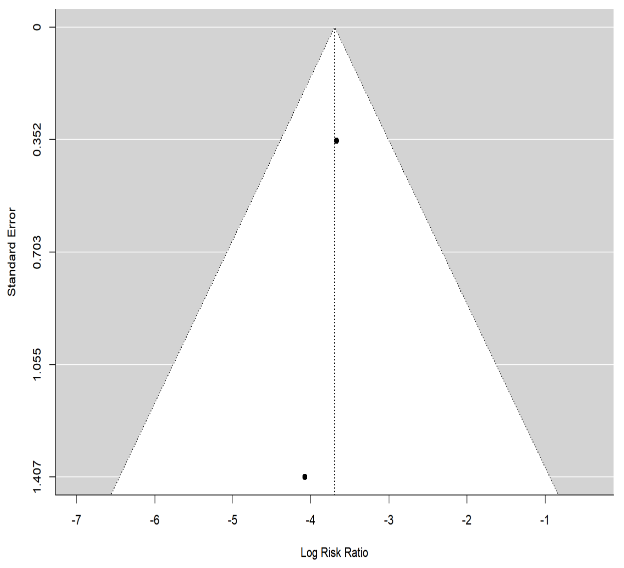

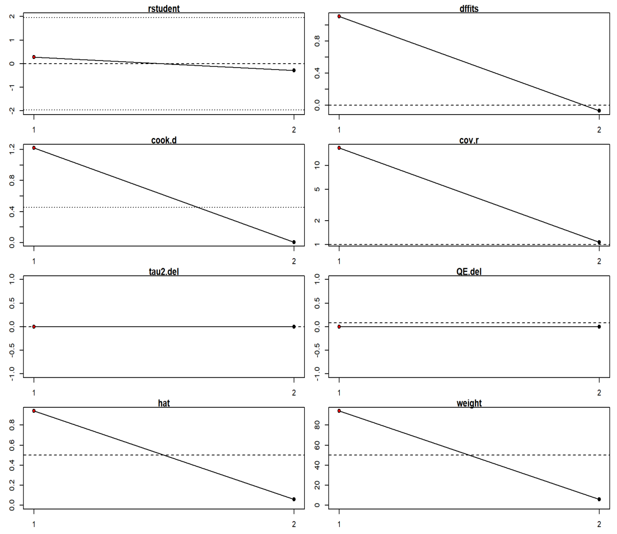

Major rescue treatment

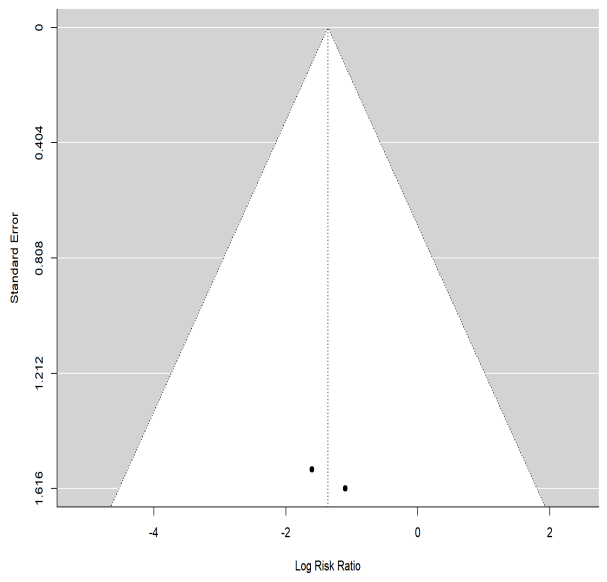

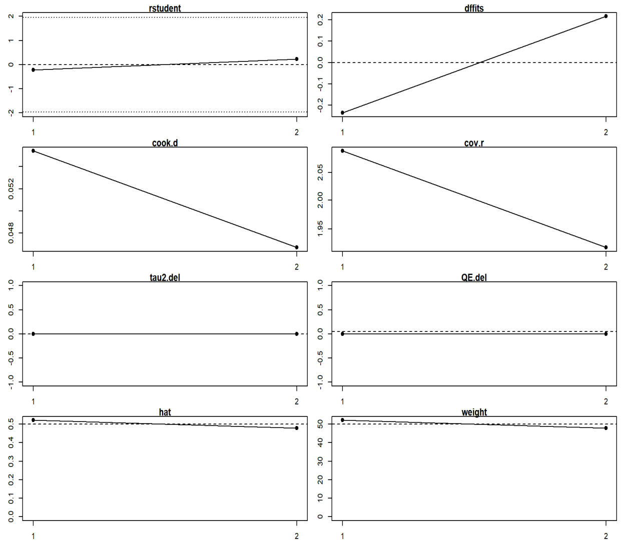

Total adverse events

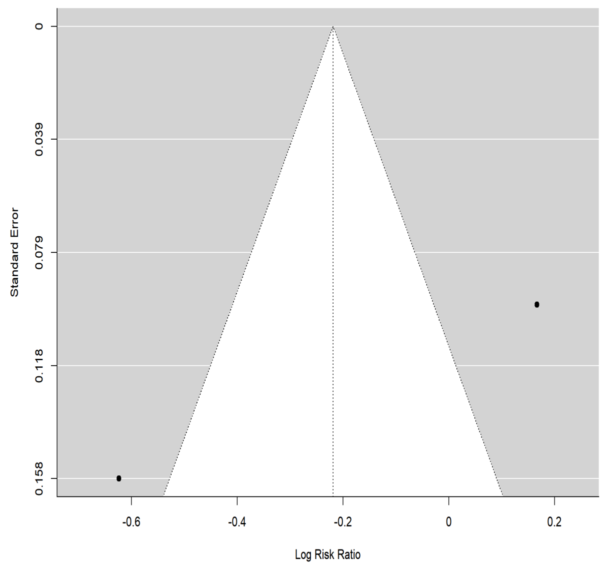

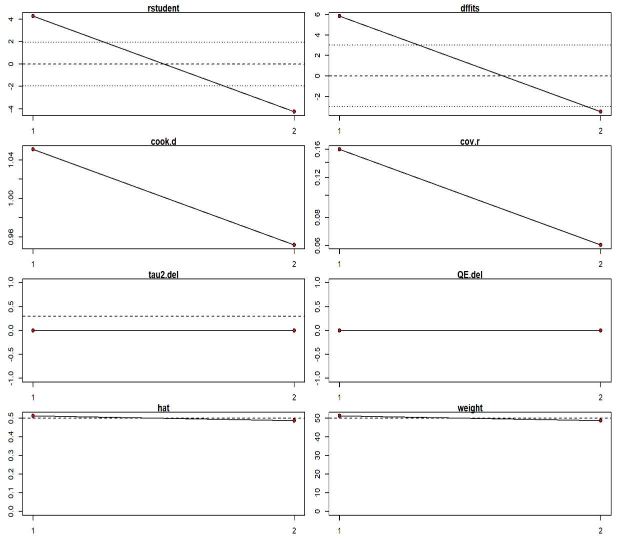

Respiratory no-hypoxemic events

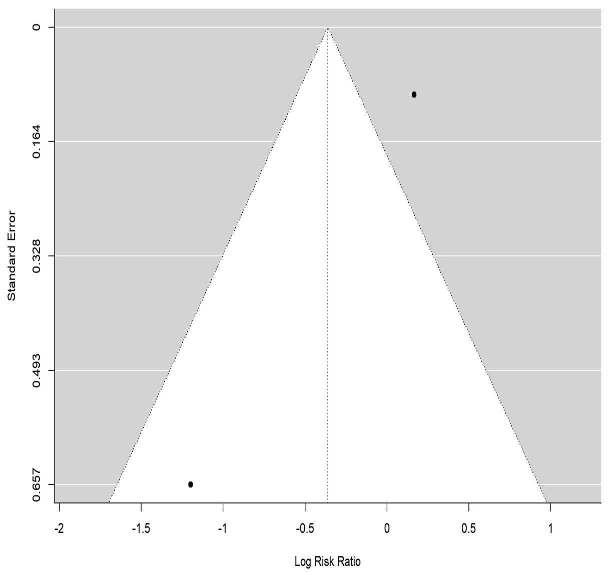

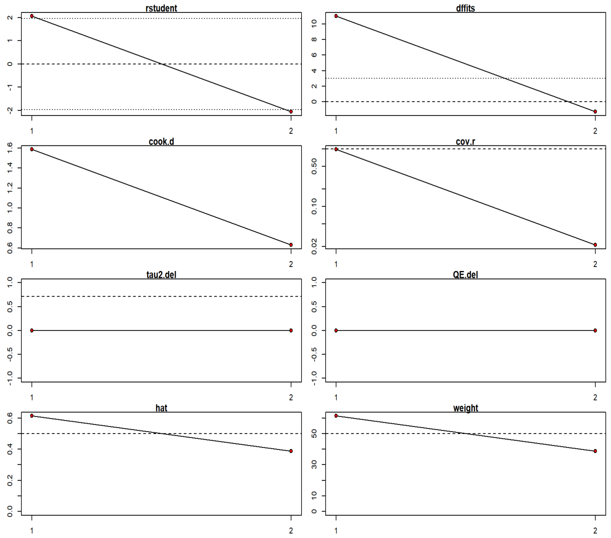

Cardiovascular events

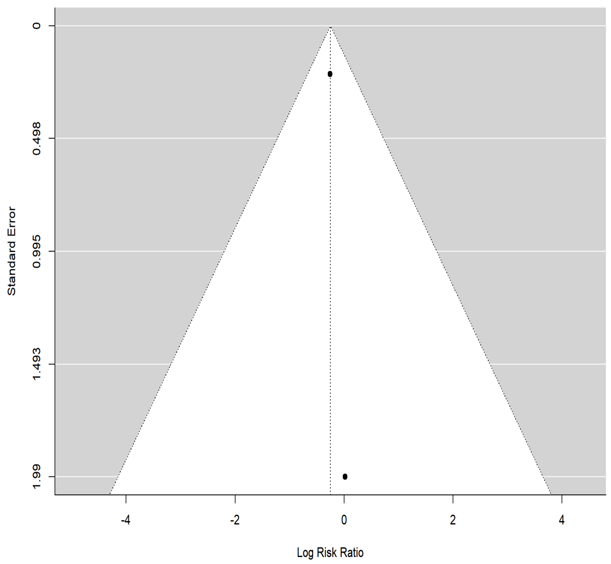

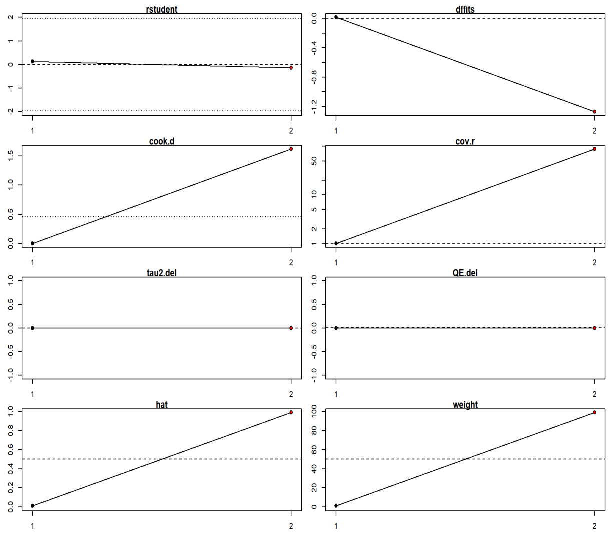

SUPPLEMENTARY MATERIAL CONTENT (SMC) 6 — Funnel plots - Outliers

Obese Patients (≥30 kg/m[2])

Hypoxic events

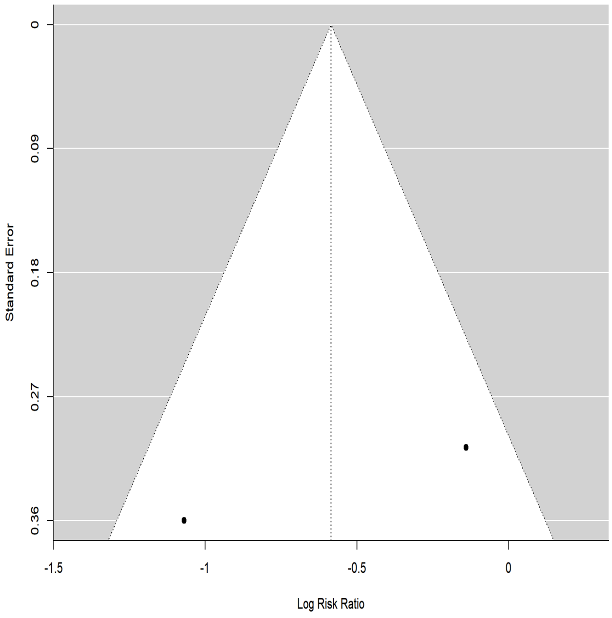

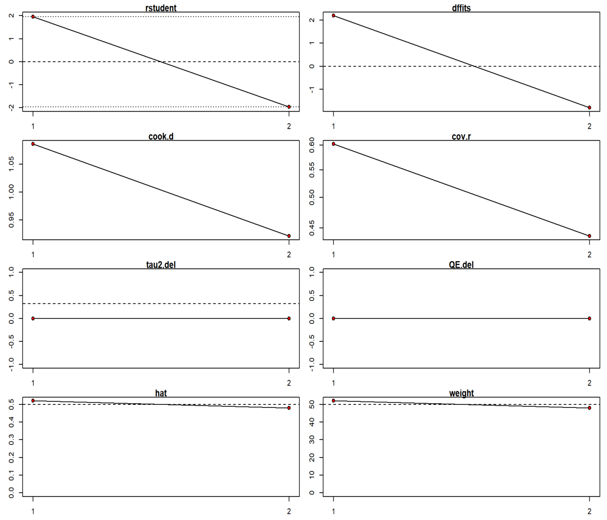

Hypoxic events $SpO_2<90\%$

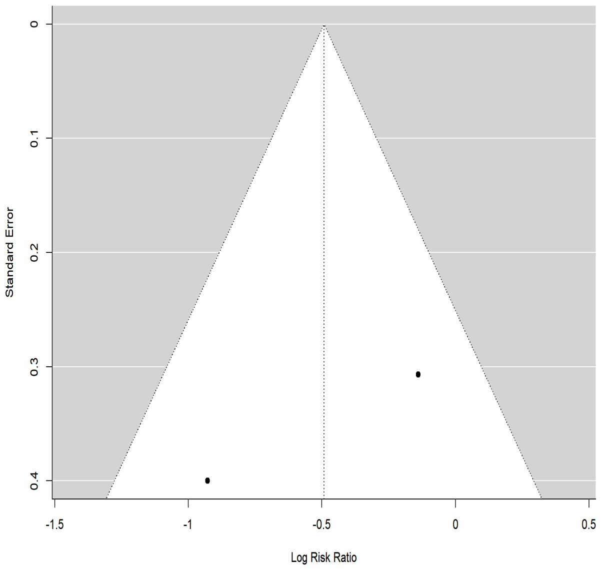

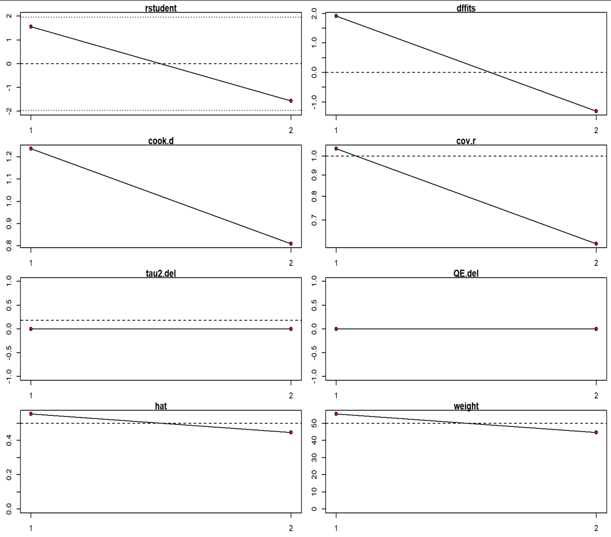

Supplement: Multimedia component 7 [file mmc7.pdf]
