# Supplementary material for: High-flow nasal oxygenation during gastrointestinal endoscopy. Systematic review and meta-analysis
Source: BJA Open. 2022 Oct 18;4:100098. doi: 10.1016/j.bjao.2022.100098 (PMC10430836; doi:10.1016/j.bjao.2022.100098)
Supplement: Multimedia component 8 [file mmc8.pdf]

Trial Sequence Analyses to detect 30% relative risk reduction in hypoxic event, with α=5% and power=80%.

Population	MIS	PC	RRR	TSMBC	DARIS	Results
**General population of patients**						
Hypoxic events	2867	0.3	0.3	No	15448	Inconclusive
Hypoxic events (SpO_2<90%)	2605	0.24	0.3	No	16975	Inconclusive
**Non-obese patients**						
Hypoxic events	2442	0.24	0.3	Yes	7595	**Firm evidence**
Hypoxic events (SpO_2<90%)	2442	0.17	0.3	No	6590	Inconclusive
**Obese patients**						
Hypoxic events	173	0.44	0.3	No	1766	Inconclusive
Hypoxic events (SpO_2<90%)	173	0.39	0.3	No	1409	Inconclusive

Hypoxic events were defined as "desaturation" (decreased peripheral capillary oxygen saturation [SpO_2]) according with the studies' endpoints observed after the induction and the maintenance of sedation for gastrointestinal endoscopy. A subgroup analysis evaluated hypoxic events defined as SpO_2<90%. General population included both non-obese and obese patients. Obesity: Body Mass Index (BMI) of ≥30 kgm^{-2}. A cumulative, sequential z score curve was constructed and used it to evaluate the adequacy of the evidence. MIS: information size in meta-analyses; PC: proportion of the event in the control group established based on literature;[9-14] RRR: Relative Risk Reduction established *a priori* based on literature;[12] TSMBC: Trial Sequential Monitoring Boundary Crossed; DARIS: Diversity Adjusted Relative Information Size. Inconclusive results indicate that further trials are likely to influence conventional meta-analysis results or that risk of random error resulting in false positive result exists.
