# Supplementary material for: High-flow nasal oxygenation during gastrointestinal endoscopy. Systematic review and meta-analysis
Source: BJA Open. 2022 Oct 18;4:100098. doi: 10.1016/j.bjao.2022.100098 (PMC10430836; doi:10.1016/j.bjao.2022.100098)

Trial Sequence Analysis to detect 30% relative risk reduction in hypoxic event, with α=5% and power=80%.

Hypoxic events defined as "desaturation" (decreased peripheral capillary oxygen saturation [SpO_2]) according with the studies' endpoints observed after the induction and the maintenance of sedation for gastrointestinal endoscopy. A subgroup analysis evaluated hypoxic events defined as SpO_2<90%. General population of patient included both no-obese and obese patients. Obese patients were defined by Body Mass Index ≥30 kg/m^2.

P_C: Proportion of the event in the control group

P_E: Proportion of the event in the treatment group

RRR: Relative Risk Reduction

Statistical analysis has been carried out using R, version 4.1.0 (2021-05-18). Specifically, Trial Sequence Analysis computations have been implemented making use of the libraries "ldbounds" and "rpact".

General population of patients

Hypoxic events according with the studies' endpoints

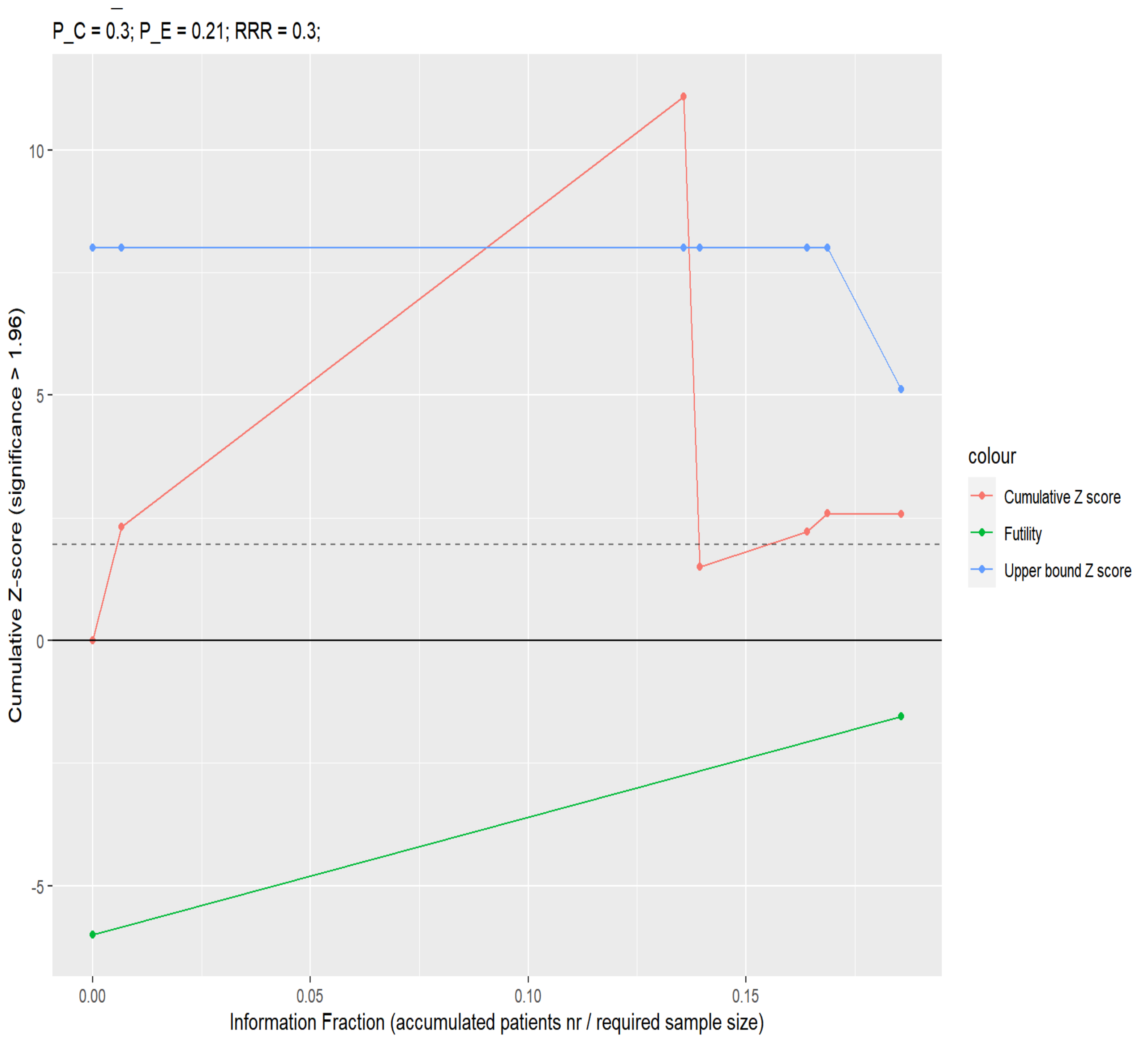

Hypoxic events SpO_2<90%

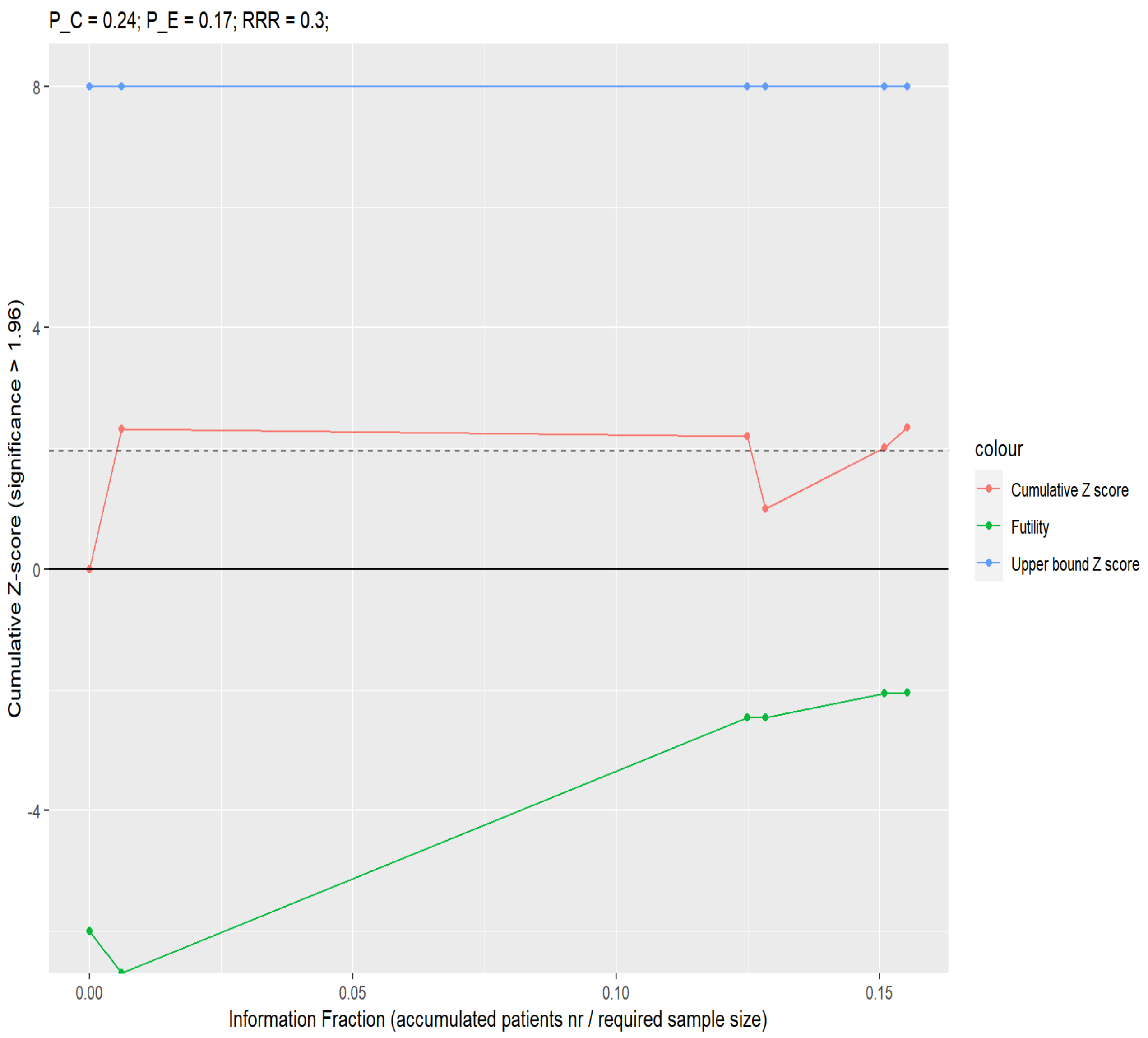

No-obese patients (<30 kg/m^2)

Hypoxic events according with the studies' endpoints

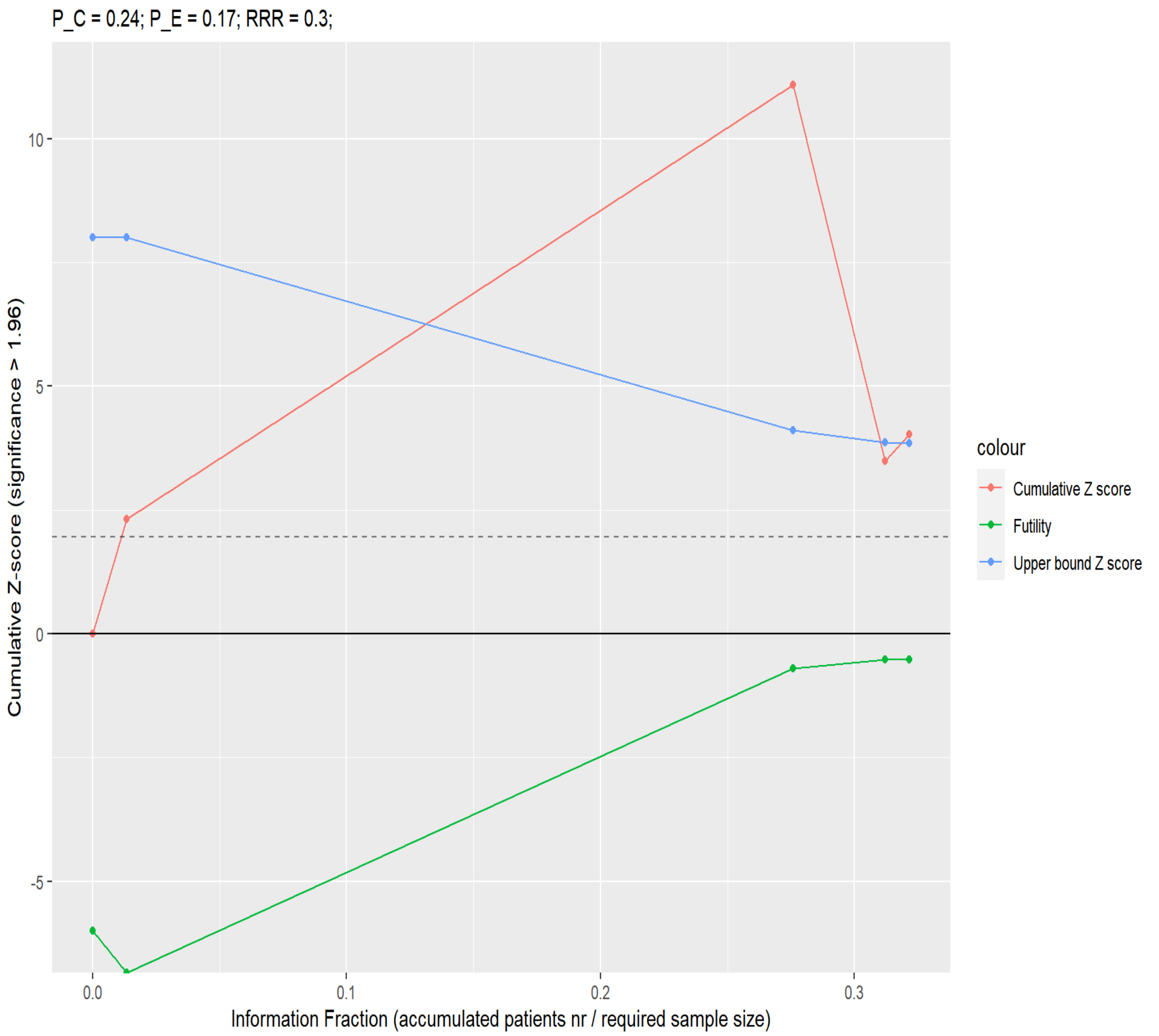

Hypoxic events SpO_2<90%

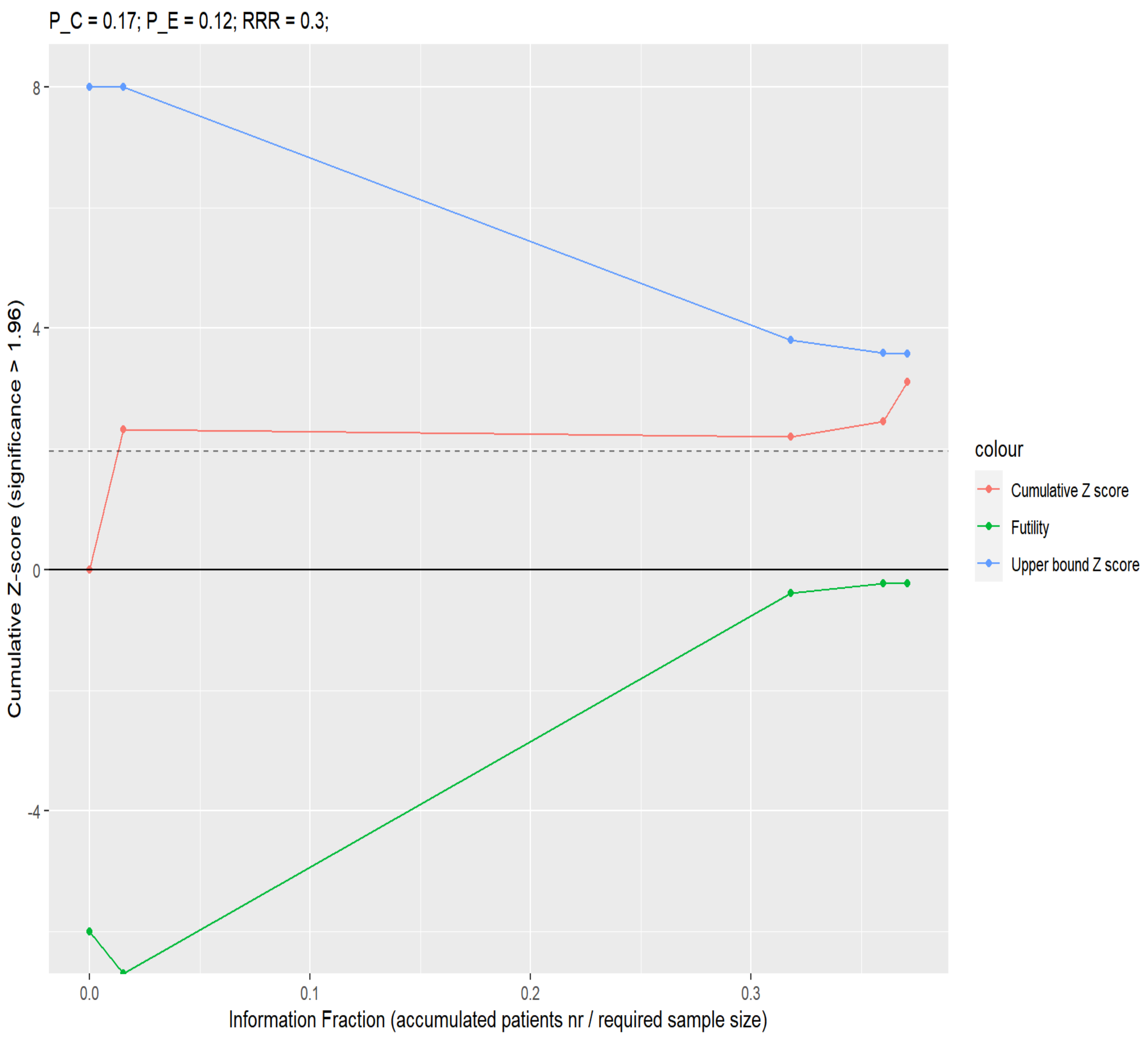

Obese patients (≥30 kg/m²)

Hypoxic events according with the studies’ endpoints

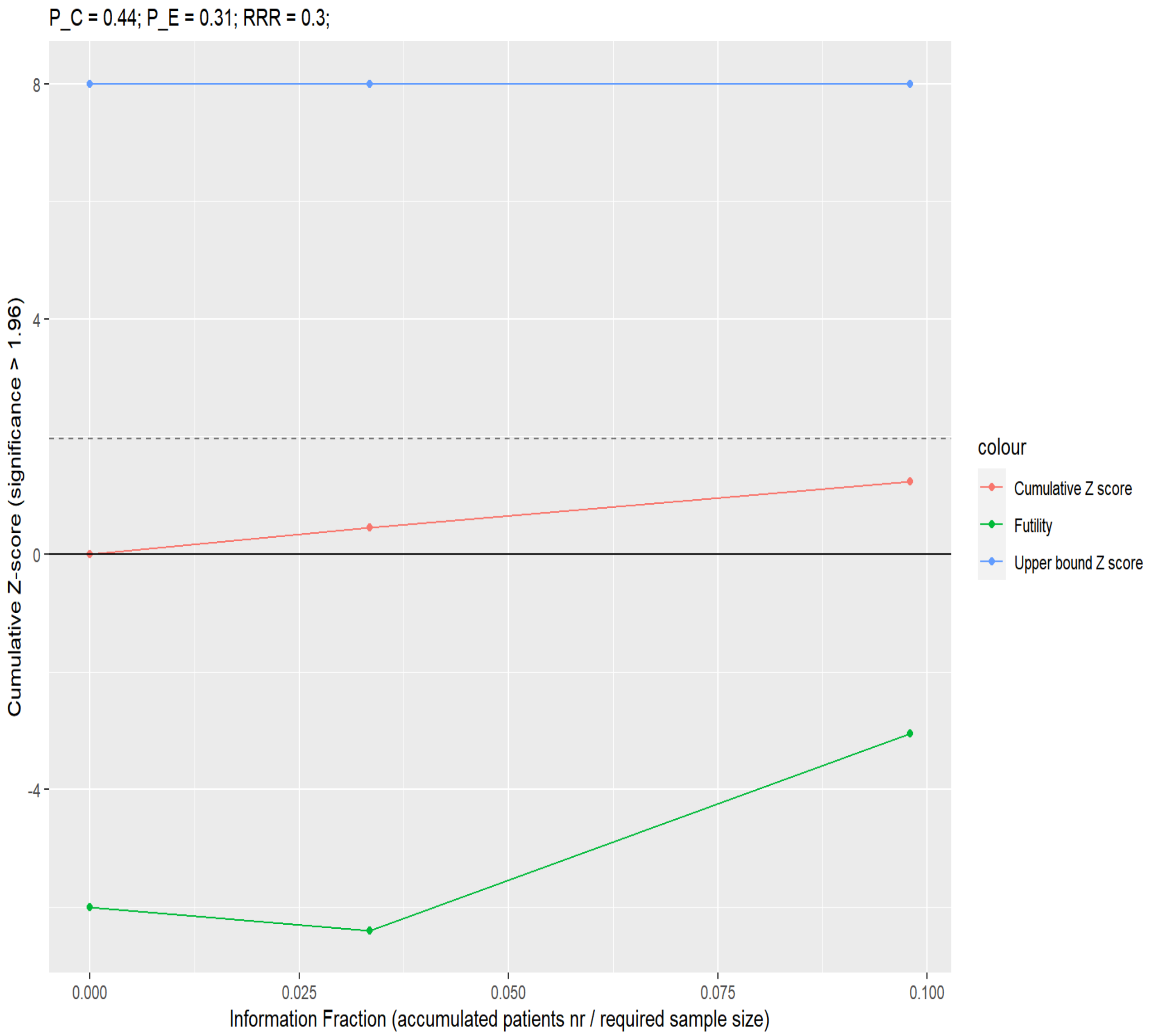

Hypoxic events SpO_2<90%

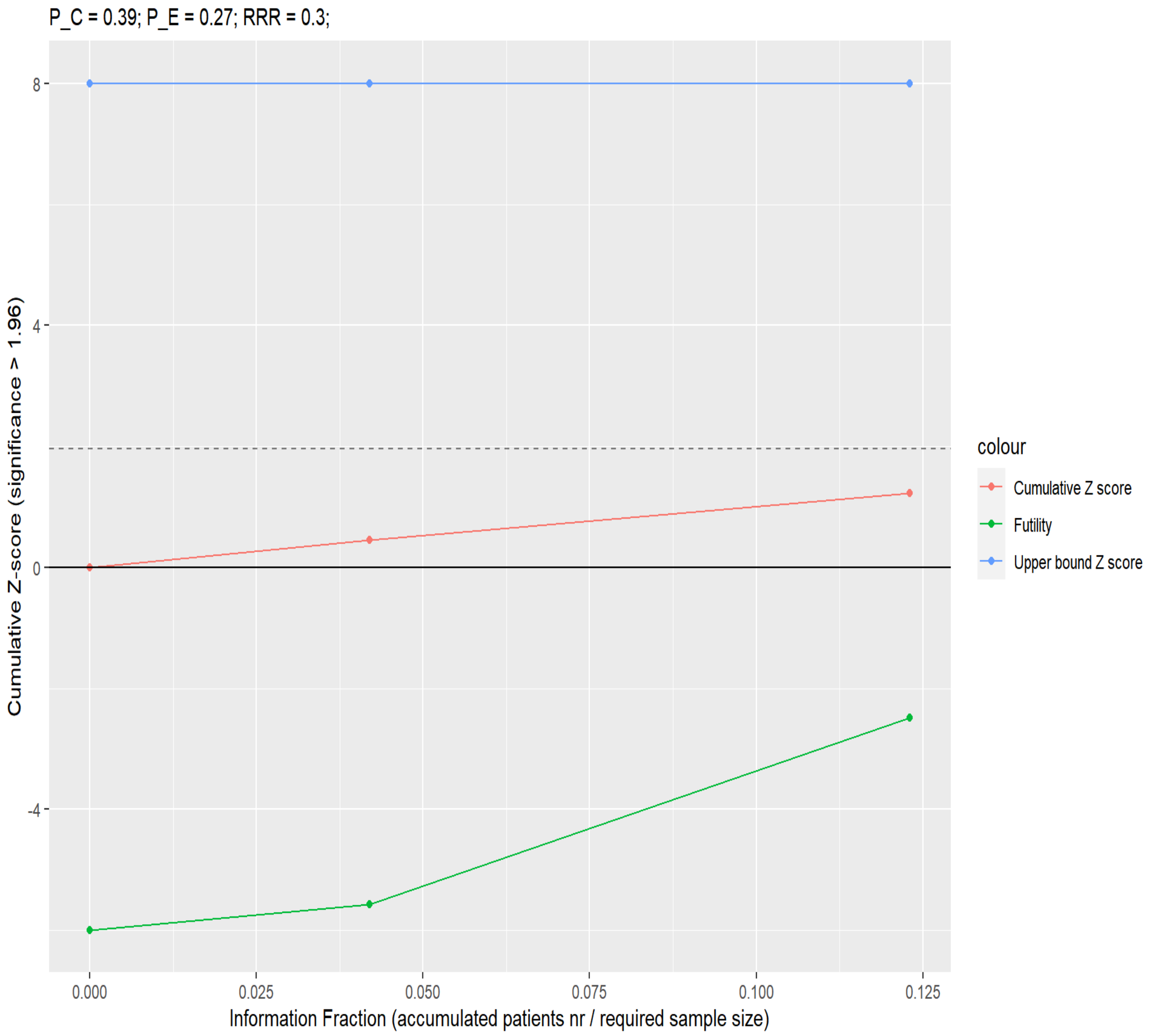

Supplement: Multimedia component 9 [file mmc9.pdf]
